# Computer-aided design of caffeic acid derivatives: free radical scavenging activity and reaction force

**DOI:** 10.1007/s00894-024-06226-2

**Published:** 2024-12-27

**Authors:** B. Carolina Morales-García, Adriana Pérez-González, J. Raúl Álvarez-Idaboy, Annia Galano

**Affiliations:** 1 Departamento de Química, Universidad Autónoma Metropolitana-Iztapalapa, Avenida Ferrocarril San Rafael Atlixco, Número 186, Colonia Leyes de Reforma 1A Sección, Alcaldía Iztapalapa, Código Postal 09310 Ciudad de Mexico, Mexico; 2 Departamento de Química, CONAHCYT - Universidad Autónoma Metropolitana - Iztapalapa, Avenida Ferrocarril San Rafael Atlixco, Número 186, Colonia Leyes de Reforma 1A Sección, Alcaldía Iztapalapa, Código Postal 09310 Ciudad de Mexico, Mexico; 3 Departamento de Física y Química Teórica, Facultad de Química, Universidad Nacional Autónoma de México, Código Postal 04510 Ciudad de Mexico, Mexico

**Keywords:** Computer-assisted design, Physicochemical properties, Reaction mechanisms, Reaction force, Free radical scavenger, Antioxidant

## Abstract

**Context:**

Antioxidants are known to play a beneficial role in human health. Caffeic acid has been previously recognized as efficient in this context. However, such a capability can be enhanced through structural modification. Thus, 3829 caffeic acid derivatives were computational designed to that purpose by adding functional groups (-OH, -SH, -OCH_3_, -COOCH_3_, -F, -CF_3_, and -N(CH_3_)(C_2_H_5_)) to its framework. Promising candidates were chosen considering drug-like behavior, toxicity, and synthetic accessibility. The best candidates, dCAF-2, dCAF-16, and dCAF-82, were identified by comparison with reference antioxidants. The thermochemistry and kinetics of their reaction with ^•^OOH are provided. The global rate coefficients were estimated to be 1.76 × 10^9^ M^−1^ s^−1^, 3.19 × 10^9^ M^−1^ s^−1^, and 1.79 × 10^9^ M^−1^ s^−1^ in aqueous solution for dCAF-2, dCAF-16, and dCAF-82, respectively. In lipid medium, their total rate coefficients were estimated to be 3.65 × 10^3^ M^−1^ s^−1^, 3.73 × 10^3^ M^−1^ s^−1^, and 8.63 × 10^4^ M^−1^ s^−1^ for dCAF-2, dCAF-16, and dCAF-82, respectively. These values allow predicting the designed caffeic acid derivatives as excellent antioxidants in both environments. The reaction forces for the main reaction path of the dCAF-2, dCAF-16, and dCAF-82 reactions with ^•^OOH were explored.

**Methods:**

Three protocols were used: (i) CADMA-Chem (computer-assisted design of multifunctional antioxidants, based on chemical properties) to quantify ADME (absorption, distribution, metabolism, and excretion) properties, toxicity and synthetic accessibility; (ii) eH-DAMA (electron and hydrogen donating ability map) tool, to identify the derivatives expected to behave as the best antioxidants; (iii) QM-ORSA (quantum mechanics–based test for overall free radical scavenging activity), to calculate the rate constants. Electronic structure calculations were performed with Gaussian 09, at the M05-2X/6–311 + g(d,p) level of theory. Both aqueous and lipid environments were considered using the SMD continuous solvation model. Intrinsic reaction coordinate (IRC) calculations, as implemented in Gaussian 09, were used to obtain the reaction force.

**Supplementary Information:**

The online version contains supplementary material available at 10.1007/s00894-024-06226-2.

## Introduction

**Scheme 1:**
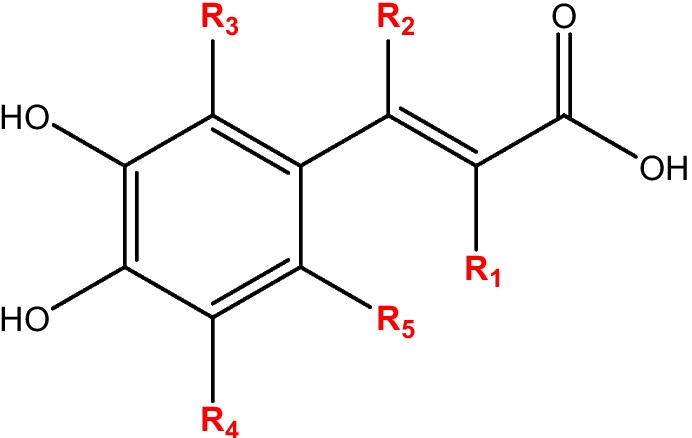
Caffeic acid (CAF) with substituted sites (R_1_ to R_5_)

Since oxidative stress (OS) has been proven to be involved in a wide variety of diseases, chemical antioxidants have become the focus of numerous investigations in the last decades. They are expected to reduce oxidative stress, inhibiting the damaging effects that free radicals, and other oxidants, may cause to important biomolecules (such as lipids, proteins, and DNA) when present in relatively large concentrations. Caffeic acid (CAF, Scheme 1), in particular, has been identified as an efficient antioxidant [1]. Moreover, it has been reported that this compound, and some of its derivatives, have a wide range of pharmacological applications. They have been proposed to be of potential use as anticancer [2–6] and anti-inflammatory [6–9] agents, in addition to their antioxidant effects [6–14]. At the same time, the potential of phenolic acids in drug discovery has been highlighted [15, 16].

With these antecedents in mind, the main goals of this work were to systematically search for caffeic acid derivatives with high potential as efficient antioxidants, identify the best candidates, and evaluate in detail the free radical scavenging activity of such candidates. Since OS has been associated with diverse neurodegenerative disorders [17–19], they might also be suitable for helping in the treatment of these diseases. In fact, the potential use of small molecules as neuroprotectors has been recently pointed out [20]. In fact, the molecules which possess a catechol group, like caffeic acid and its designed derivatives (Scheme 1), are targets of catechol-o-methyl transferase (COMT), an enzyme that metabolizes neurotransmitters. Thus, COMT inhibitors work by blocking or reacting with this enzyme and therefore protect neurotransmitters [21–23].

Some studies have also been conducted to investigate the therapeutic potential of caffeic acid against beta-amyloid (Aβ1-42)–induced oxidative stress, related to the Alzheimer’s disease, and memory impairments. They indicated that caffeic acid administration improves spatial learning, memory, and cognitive abilities in mice with Alzheimer’s disease [24]. There are related reviews on the neuroprotective potential of the main bioactive components of coffee, including caffeic acid [25]. There are other studies confirming the neuroprotective activity of caffeic acid [26–29]. It has also been suggested that caffeic acid exerts an antioxidant effect, which was evidenced by the decrease in the levels of oxidative stress markers throughout the brain. The data in reference [27] suggest that caffeic acid can prevent acute neurodegenerative changes induced by neuroinflammation and probably long-term neurodegenerative changes.

With all this in mind, in this work, new derivatives of caffeic acid were designed by using its molecular framework as a starting point. They are expected to maintain the protective properties of the parent molecule, and, in addition, the inclusion of functional groups can enhance and diversify the activity. Such a multifunctionality is expected to be useful in the treatment of multifactorial diseases such as Alzheimer’s and Parkinson’s.

The search for new caffeic acid derivatives was made using a computer-assisted protocol known as CADMA-Chem (computer-assisted design of multifunctional antioxidants, based on chemical properties) [30–32] by incorporating functional groups (-OH, -SH, -OCH_3_, -COOCH_3_, -F, -CF_3_, and -N(CH_3_)(C_2_H_5_)) in the R_1_–R_5_ sites of the parent molecule (Scheme 1). From a chemical point of view, some of the reasons why these functional groups have been selected are the following. -COOCH_3_ increases the interaction with the receptor sites in the enzymes. -OH and -SH favor the antioxidant activity through hydrogen atom transfer to the free radical. -OCH_3_, -N(CH_3_)(C_2_H_5_), -OH, and -SH favor modifying the pKa values and, consequently, the fraction of the neutral species at physiological pH (i.e., promoting passive crossing through biological membranes). In addition, these functional group usually increase chelation of metal ions, an important mechanism to inhibit the formation of the OH free radical via Fenton-like reactions. Fluorinated moieties, such as -F and -CF_3_ also increase passive crossing through the lipid membrane of the cell.

ADME properties (adsorption, distribution, metabolism, and excretion), toxicity, and synthetic accessibility were investigated as well as the thermochemistry and kinetics related to the antioxidant potential of the most promising candidates.

## Methods

### Pharmacological behavior

It is important to clarify that the analysis carried out in the following sessions (reference set, ADME properties, toxicity, synthetic accessibility, selection score, and elimination score) involve QSAR computational strategies, carried out using SMILE identifiers for the investigated chemical structures. For electronic calculations, estimation of pKa values, reactivity descriptors, rate constants, and the reaction force, a chemical modeling of the corresponding chemical structures in 3D has been carried out with the help of the Gaussview program.

#### Reference set

To put into perspective the relevance of the calculated data, a reference set of molecules was used. It is composed by 54 chemical compounds that are already in use as neuroprotectors (Table S2, Supporting Information). For all of them, and the newly designed derivatives, the chemical descriptors were calculated, i.e., ADME properties, toxicity, and synthetic accessibility (Table S3, Supporting Information). The average values of each property, considering the 54 reference molecules, were used to estimate the selection score (*S*^*S*^) of this reference set. That *S*^*S*^ was then used as a threshold to identify the best candidates among all designed caffeic acid derivatives, i.e., only those with *S*^*S*^ values that surpass that of the reference set are considered as viable candidates. More details on this score are provided below.

#### ADME properties, toxicity and synthetic accessibility

The estimated ADME properties were number of donors in H-bridge interactions (HBD), number of acceptors in H-bridge interactions (HBA), molecular weight (MW), octanol/water partition coefficient (log P), molar refractivity (MR), number of non-hydrogen atoms (AtX), number of rotatable bonds (RB), and polar surface area (PSA). They all were obtained using RDKit [33]. These descriptors are necessary to investigate if the designed caffeic acid derivatives (dCAF) satisfy Lipinski’s rule of five [34], the Ghose’s rule [35], and the Veber criteria [36].

The toxicity of all dCAF was also investigated. To that purpose the, Toxicity Estimation Software Tool (TEST) (https://www.epa.gov/chemical-research/toxicity-estimation-software-tool-test), version 4.1., was used. This software makes predictions based on quantitative structure–activity relationships (QSAR). In the present investigation, the consensus method was used to calculate the LD_50_ descriptor and the Ames mutagenicity.

The AMBIT-SA software (https://ambit.sourceforge.net/reactor.html) was used to estimate the synthetic accessibility (SA) of the designed compounds. The values are scaled and weighted to provide a number in the range of 1 to 100 (the larger the value, the easier the synthesis).

#### Selection score (SS) and elimination score (S.^E^)

The selection score (*S*^*S*^) allows assigning a single number to each molecule, and it was designed in such a way that higher numbers correspond to better candidates. It includes three terms that account for the desired ADME properties (*S*^*ADME*^) for oral drugs, low toxicity (*S*^*T*^), and accessible synthesis (*S*^*SA*^). More details on the *S*^*S*^ formulations are provided in Supporting Information.

To verify if any designed caffeic acid derivative significantly deviates from the average value of the reference set, in any of the analyzed properties, the elimination score (*S*^*E*^) was used. The details on this score are also provided in Supporting Information.

### Antioxidant behavior

Once the ADME, toxicity, and synthetic accessibility properties were estimated, the selection and elimination scores were calculated from them for each of the designed caffeic acid derivatives. These scores were then used to identify the best candidates. The chemical modeling and electronic structure calculations were carried out using the GaussView and Gaussian programs. More details on the methodology used to determine the primary antioxidant capacity of caffeic acid derivatives identified as the most promising candidates are provided next.

#### Electronic calculations

The Gaussian 09 package of programs [37] was used for the electronic calculations. Local minima were confirmed by the absence of imaginary frequencies. Unrestricted calculations were used for open-shell systems. All geometry optimizations and frequency calculations were carried out using the density functional theory (DFT), with the M05-2X functional [38], the 6–311 + G(d,p) basis set, and SMD [39] solvation model using water and pentylethanoate to mimic a polar and no polar environment, respectively. SMD is considered a universal solvation model due to its applicability to any charged or uncharged solute in any solvent or liquid medium for which a key descriptors are known. M05-2X is a global hybrid exchange–correlation GGA approach to DFT designed for thermochemistry, kinetics, and noncovalent interactions. It has also been recommended for calculating reaction energies involving free radicals [40–43]. Furthermore, the M05-2X functional has been widely used for estimating *p*Ka values, bonding dissociation energies, and the free radical scavenging activity of several antioxidant molecules.

#### Estimation of pKa values

The acid constants (*p*Ka) were calculated for the derivatives identified as promising antioxidant candidates, according to their *S*^*S*^ values. The fitted parameters approach (FPA) [44, 45] was used to do for that purpose:

1 $${p\text{K}a}_{exp}={m\Delta G}_{BA}+{C}_{0}$$

where* m* and *C*_*0*_ are the parameters obtained from linear fittings using experimental data. They are currently available at numerous levels of theory for phenols, amines, carboxylic acids, and thiols [44, 45]. The values of this parameters that correspond to the level of theory used in this investigation are provided in Table 1.

**Table 1:** Values of the *m* and *C*_0_ parameters, at M05-2X/6–311 + G(d,p) level of theory, for different functional groups

Functional group	*m*	*C*_0_	Ref
Thiol	0.357	− 94.639	[45]
Phenol	0.316	− 81.497	[44]
Carboxylic acid	0.356	− 94.380	[44]
Amine	0.464	− 121.000	[44]

It has been demonstrated that the *p*K_*a*_ values calculated with the FPA approach deviate from experiments by less 0.2 *p*K_*a*_ units, in terms of mean unsigned errors.

#### Reactivity descriptors

The ionization energies (IE) and bond dissociation energies (BDE) were estimated to analyze the likeliness of the designed compounds to act as antioxidants through single electron transfer (SET) and formal hydrogen transfer (*f*-HAT) chemical routes (Table S6). For the calculations of BDE, all sites that act as H donors were considered, including the new added functional groups (-OH, -SH, -OCH_3_, -OOCH_3_, -F, -CF_3_, and -N(CH_3_)(C_2_H_5_)) in R_1_–R_5_ (Scheme 1).

#### Rate constants

The rate constants (k) were calculated using conventional transition state theory (TST) [46, 47] and 1 M standard state (Eq. 2):

2 $$k=\sigma k\frac{{k}_{B}T}{h}{e}^{-({\Delta G}^{\ne })/RT}$$

where *k*_*B*_ and *h* are the Boltzmann and Planck constants, Δ*G*^*≠*^ is the Gibbs free energy of activation or energetic barriers, *σ* represents the reaction path degeneracy, accounting for the number of equivalent reaction paths, and *κ* accounts for tunneling corrections. The tunneling corrections defined as the Boltzmann average of the ratio of the quantum and the classical probabilities were calculated with zero curvature (ZCT)approach [48].

For electron transfer reactions, the barriers were estimated using the Marcus theory [49]. Some of the calculated rate constants were found to be close to the diffusion-limit. Accordingly, the apparent rate constant (*k*_app_) cannot be directly obtained from TST calculations. In the present work, the Collins–Kimball theory was used to include the rate limit imposed by diffusion (Eq. 3) [50]:

3 $${k}_{\text{app}}=\frac{{k}_{D}{k}_{\text{act}}}{{k}_{D}+{k}_{\text{act}}}$$

where *k*_act_ is the thermal rate constant, obtained from TST calculations (Eq. 1), and *k*_*D*_ is the steady-state Smoluchowski [51] rate constant for an irreversible bimolecular diffusion-controlled reaction (Eq. 4):

4 $${k}_{D}={4\pi RD}_{AB}{N}_{A}$$

where *R* denotes the reaction distance, *N*_*A*_ is the Avogadro number, and* D*_*AB*_ is the mutual diffusion coefficient of the reactants A and B. *D*_*AB*_ has been calculated from *D*_*A*_ and *D*_*B*_ according to reference [52], while *D*_*A*_ and *D*_*B*_ have been estimated from the Stokes–Einstein approach (Eq. 5) [53, 54]:

5 $$D=\frac{{k}_{B}T}{6\pi \eta a}$$

where *k*_*B*_ is the Boltzmann constant, *T* is the temperature, *η* denotes the viscosity of the solvent, in our case water (*η* = 8.91 × 10^−4^ Pa s) and pentylethanoate (*η* = 8.62 × 10^−4^ Pa s); and *a* is the radius of the solute.

#### The reaction force

Considering the importance of a detailed kinetic study to determine the primary antioxidant activity of the studied compounds, it is crucial to characterize the stationary points including reactants, transition states, and products. The reaction force (RF) was used to that purpose, since it provides important insights on the chemical transformations that takes place as the reaction progresses.

Intrinsic reaction coordinate (IRC) calculations were performed to establish the path of minimum potential energy linking the transition state to reactants and products [55–57]. The negative of the first derivative of the potential energy with respect to position along the IRC gives was used to calculate the reaction force:

6 $$\text{RF}(\text{IRC})=-\frac{\partial \text{V}(\text{IRC})}{\partial \text{IRC}}$$

As previously mentioned by Politzer and Toro-Labbé [55], a key point that is brought out and emphasized by $\text{RF}(\text{IRC})$ is that an IRC is characterized by three well defined points, $\alpha$, $\beta$, and $\gamma$. Thus, the transition state is only one of the three significant milestones along the reaction path.

## Results and discussion

### Systematic computational design

**Fig. 1:**
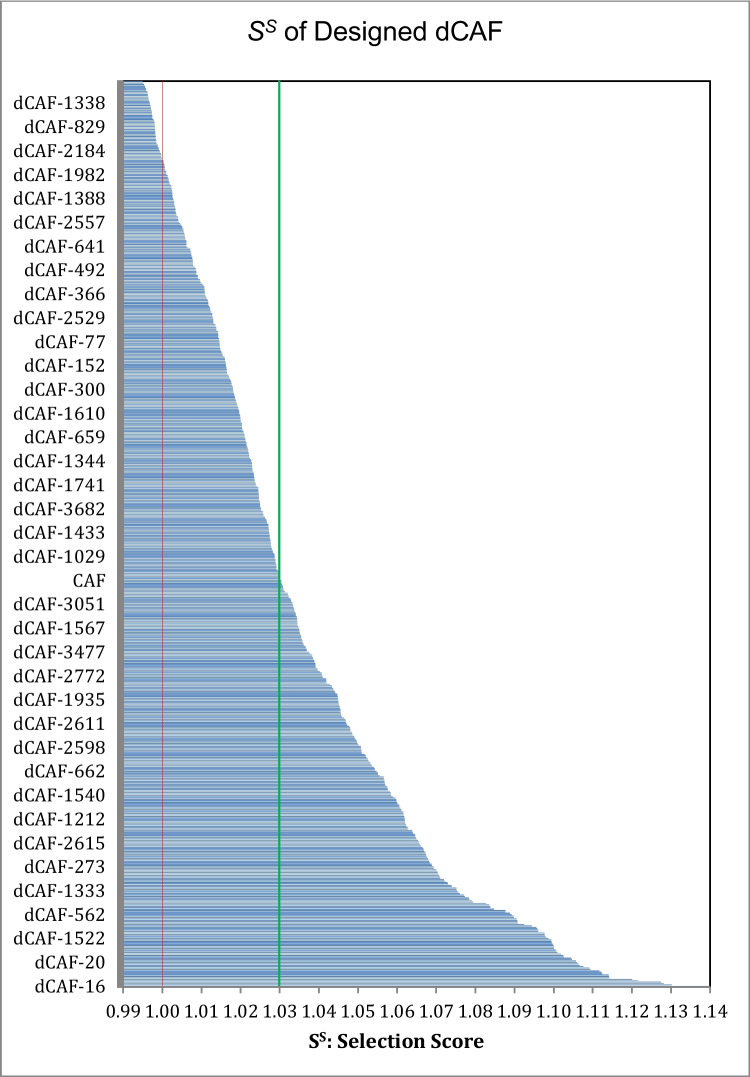
Selection score (*S*^*S*^) for the designed caffeic acid derivatives

**Scheme 2:**
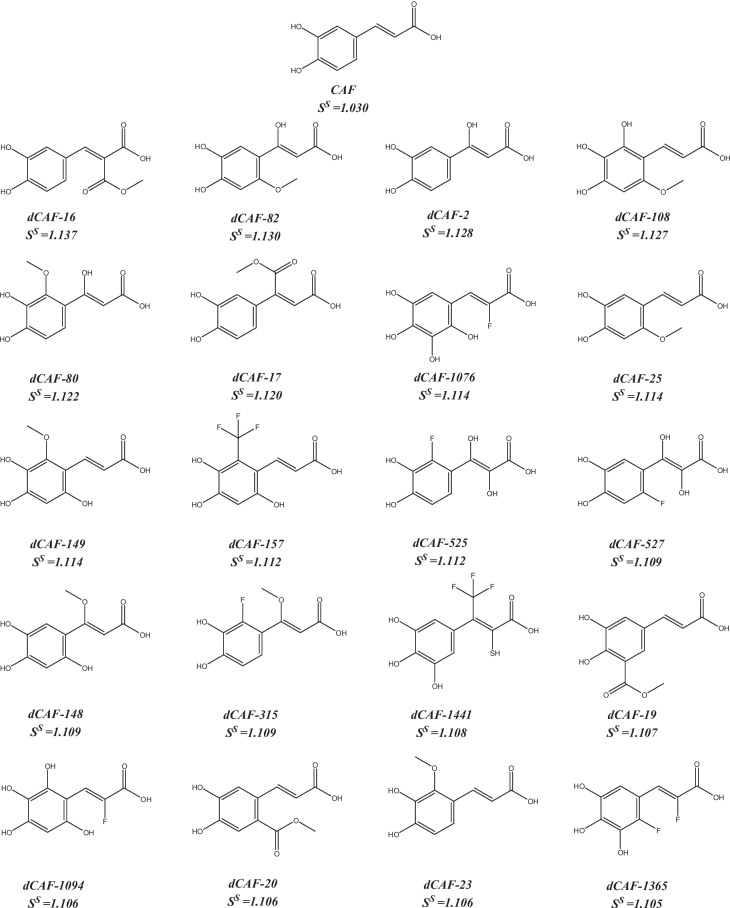
Structures of caffeic acid and the best 20 derivatives

From the structure of caffeic acid, designed derivatives were obtained by incorporating the following functional groups: -OH, -SH, -OCH_3_, -COOCH_3_, -F, -CF_3_, and -N(CH_3_)(C_2_H_5_) in the R_1_–R_5_ sites shown in Scheme 1. From this design strategy, 3829 caffeic acid derivatives (dCAF) were obtained. Their selection scores (*S*^*S*^) are shown in Fig. 1 and in Fig. S1. The red line in this figure (*S*^*S*^ = 1.0) corresponds to the set of reference compounds. Caffeic acid, on the other hand, has *S*^*S*^ equal to 1.03 (green line in Fig. 1), which tells us that its drug-like behavior (according to the Lipinski, Ghoose, and Veber rules) is better than the average of the 54 neuroprotectors, already in use, that are included in the reference set. The 645 dCAF derivatives with *S*^*S*^ values larger than both, the average of the reference set and the that of caffeic acid, are considered as viable candidates to act as oral antioxidants. Among these 645 candidates, the 20 with the highest selection scores were selected for the next stage of the investigation. Their structures are shown in Scheme 2.

**Fig. 2:**
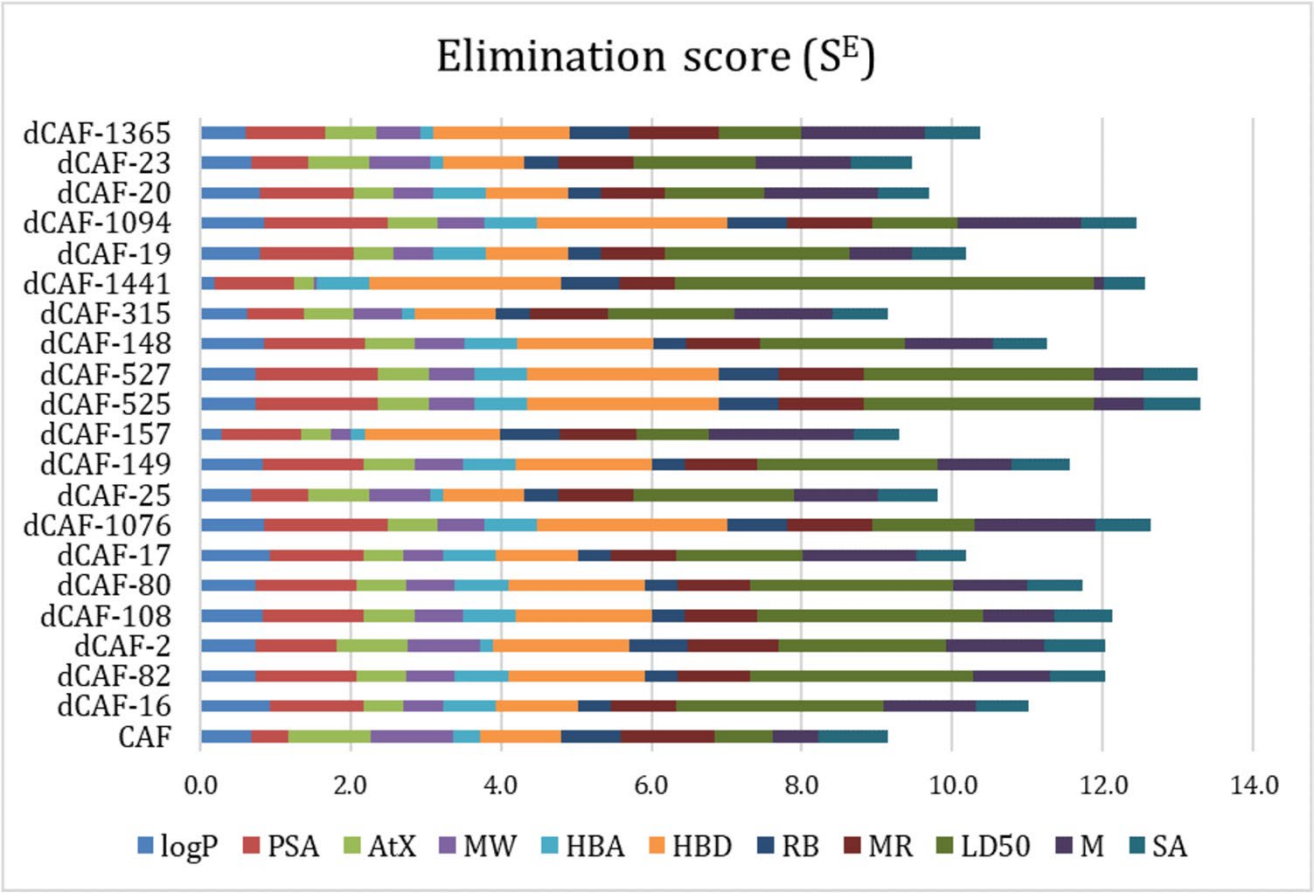
Elimination scores (*S*^*E*^) for the best 20 designed dCAF

The values of the ADME properties, toxicity, and synthetic accessibility of these 20 derivatives are provided in Table S4, as well as their *S*^*S*^ values. Their elimination scores (*S*^*E*^) are shown in Fig. 2, while the values are reported in Table S5, were caffeic acid is also included for comparison purposes. The main deviations were found in HBD and LD_50_. The dCAFs with the largest deviations in HBD have values equal to 5, indicating that they still fall within the range proposed by the rules of bioavailability and permeability (HBD ≤ 5). The derivatives that deviate more in LD_50_ have values much higher than the average value for the reference drugs (1142.87 mg/kg), which indicates that it is a desirable deviation since they are predicted to be less toxic than the reference set.

**Scheme 3:**
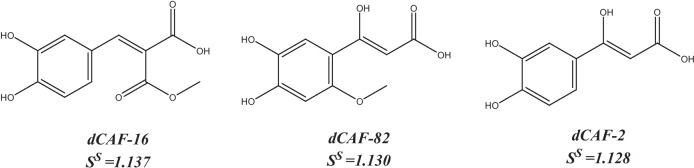
Designed dCAF with the highest selection scores (*S*.^*S*^)

The designed derivatives of caffeic acid with the highest selection scores are dCAF-16 (*S*^*S*^ = 1.137), dCAF-82 (*S*^*S*^ = 1.130), and dCAF-2 (*S*^*S*^ = 1.128). Their structures are shown in Scheme. 3. These dCAFs were obtained by adding the –COOCH_3_ group in the R_1_ site (dCAF-16), –OH, and –OCH_3_ groups in the R_2_ and R_5_ sites (dCAF-82), and –OH group in the R_2_ site (dCAF-2). They are predicted as the most likely derivatives to behave as bioavailable, free of permeability issues, easily synthesized, and they low-toxicity compounds among the designed ones.

### Estimation of pKa values

For the three of them conformational analyses were carried out, at the M052X/6–311 + G(d,p) level of theory, using the SMD continuous model and water as a solvent (Table S1, Supporting Information). After identifying the conformers lowest in energy, the deprotonation route and theoretical *p*Ka’s values were obtained. Their deprotonation mechanisms are shown in Scheme S1, and their distribution diagrams are provided in Fig. S2, Supporting Information, while the *p*Ka values are reported in Table 2 together with the molar fraction (*Mf*) of each species in aqueous solution at *p*H = 7.4 (physiological conditions). For all of them the mono-anion and di-anion species have significance molar fraction under these conditions. However, the neutral species also was considered in the calculations of the reactivity descriptors (IE and BDE) for comparison purposes.

**Table 2:** *p*Ka (± 0.2) values and molar fractions (*Mf*) for selected designed dCAF, at physiological *p*H, in aqueous solution

	*p*Ka (± 0.2)				*Mf*		
dCAF	*p*Ka1	*p*Ka2	*p*Ka3	*p*Ka4	Neutral	Mono-anion	Di-anion
dCAF-16	3.33	7.58	11.92		0.0001	0.6027	0.3973
dCAF-82	3.72	8.11	12.25	12.43	0.0002	0.8359	0.1639
dCAF-2	3.66	7.94	12.22	13.33	0.0001	0.7764	0.2235

### Reactivity descriptors

**Fig. 3:**
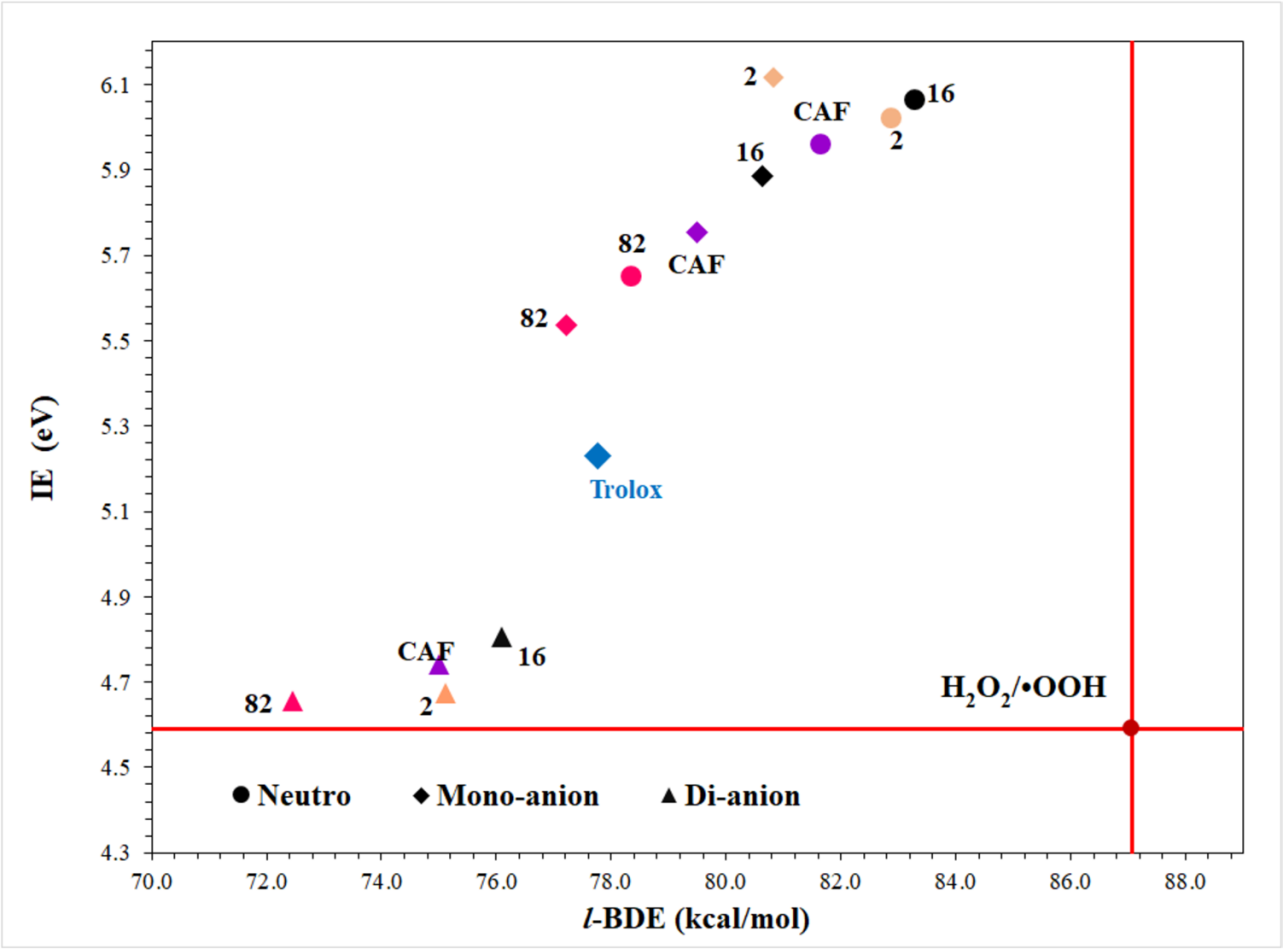
eH-DAMA map for dCAF-16, dCAF-82, and dCAF-2

Figure 3 shows the electron and hydrogen donating ability map for antioxidants (eH-DAMA) that is composed using both BDE and IE values (Table S7). Caffeic acid and Trolox were included in this map for comparison purpose. The oxidant H_2_O_2_/O_2_^•−^ pair was included because the OOH radical is considered as the oxidant counterpart of the investigated molecules. The eH-DAMA is a graphical aid that help quickly identify chemical species capable of donating one H atom or one electron to the oxidants by just looking at their relative location on the map.

Derivative dCAF-82 is located at the left of caffeic acid and Trolox (i.e., its BDE is lower than that of HOOH) which means that it is a better H donor than the parent molecule and the reference antioxidant, regardless of its deprotonation state. On the other hand, derivatives dCAF-2 and dCAF-16 do not surpass caffeic acid nor Trolox as H donor. However, all of them are predicted as efficient for deactivating the OOH radical through H donation. Contrarily, all the species on the eH-DAMA are located above the H_2_O_2_/O_2_^•−^ pair (i.e., they have higher IE than OH^−^), including Trolox and caffeic acid. Thus, none of them are expected to deactivate ^•^OOH via SET.

### Thermochemistry and kinetics

**Scheme 4:**
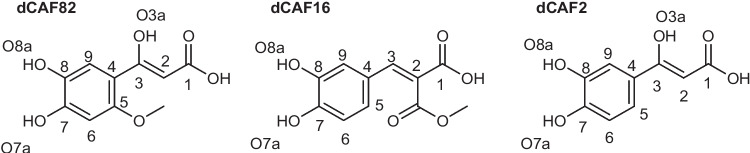
Structure and site numbering of dCAF-2, dCAF-16, and dCAF-82

Thermochemistry and kinetics of every possible chemical route involved in the reactions of dCAF-82, dCAF-16, and dCAF-2 with the OOH free radical were explored, following the results obtained from the eH-DAMA map that allowed identified dCAF-82 derivative (Scheme 4) as the most promising one as antioxidant. They are consistent with three different mechanisms: single electron transfer (SET), hydrogen atom transfer (HT), and radical adduct formation (RAF), as the QM-ORSA (quantum mechanics–based test for overall free radical scavenging activity) protocol suggests for this kind of compounds. However, only for dCAF-82, the three different mechanisms were analyzed. Such an analyses allowed to rule out the RAF route as a significant one for this kind of compounds. Consequently, for dCAF-2 and dCAF-16 only SET and f-HAT mechanisms were considered. The structures and site numbering of the CAF derivatives are provided in Scheme 4. The Gibbs free energies (Δ*G*, kcal/mol) for the reactions of the neutral, mono-anion, and di-anion species of dCAF-82, dCAF-16, and dCAF-2 with ^•^OOH, in water and pentylethanoate solvents, are reported in Table 3.

**Table 3:** Gibbs free energies of reaction (Δ*G*, kcal/mol) for neutral, mono-anion, and di-anion species of dCAF-2, dCAF-16, and dCAF-82 with ^•^OOH, in water (W) and pentylethanoate (PE), at 298.15 K

dCAF-82	PE		Water	
	Neutral	Neutral	Mono-anion	Di-anion
*f*-HAT				
O3a	10.52	8.24	2.59	− 0.38
O7a	− 7.44	− 6.49	− 8.14	––
O8a	− 9.88	− 8.89	− 9.99	− 15.05
RAF				
C2	9.90	7.66	7.72	4.37
C3	10.55	8.08	8.34	4.42
C4	16.93	13.92	17.33	9.70
C5	9.77	8.88	12.86	6.57
C6	15.17	14.85	18.64	11.47
C7	8.35	8.57	12.72	10.34
C8	11.13	9.26	12.65	4.43
C9	13.59	11.64	17.34	11.01
SET	––	25.06	22.24	1.48
dCAF-2				
*f*-HAT				
O3a	11.81	6.38	3.90	3.57
O7a	− 3.70	− 4.23	− 6.34	––
O8a	− 2.78	− 3.41	− 4.83	− 12.09
SET	––	32.17	33.37	3.37
dCAF-16				
*f*-HAT				
O7a	− 3.47	− 3.87	− 6.45	––
O8a	− 1.77	− 2.73	− 4.41	− 10.89
SET	––	33.67	29.70	4.24

From the thermodynamical data, the RAF mechanism was excluded as viable, since all the reaction channels were found to be endergonic, regardless of the solvent and of the deprotonation state of dCAF-82. On the contrary, *f-*HAT from the phenolic groups (O7a and O8a sites, Scheme 4) are predicted as significantly exergonic both in pentylethanoate and in aqueous solution for all the acid–base species of the dCAF selected. The Gibbs free energies of reaction corresponding to the SET mechanism, in aqueous solution, are all positive, indicating that such processes are not spontaneously feasible at room temperature for the dCAF selected. The thermochemistry of the SET and *f*-HAT reactions supports the predictions from the eH-DAMA plot.

The energy barriers and rate constants corresponding to those reaction paths identified as thermochemically viable are reported in Tables 4 and 5. The structures and imaginary frequencies of the associated transition states are provided in Fig. S3 to S5, Supporting Information. The *f*-HAT path involving site O8a in the dCAF-2, dCAF-16, and dCAF-82 di-anions were found to be barrier-less and, consequently, diffusion controlled. The energy profiles, demonstrating that this process takes place this way, are provided in Fig. S6 to S8, Supporting Information. The barriers of all the other reaction channels range from 6.86 to 17.77 kcal/mol. For neutral species, the rate constants increase with the solvent polarity for the three analyzed designed dCAF in the exergonic reaction, with the exception of O7a site, for which the reaction barrier is lower in pentylethanoate than in water. This inversion is caused by the tunneling correction (*κ*), i.e., *κ*_water_ > > *κ*_pentylethanoate_ (Table S8, Supporting Information). Regarding acid–base equilibria, a systematic trend was found. For dCAF-2, dCAF-16, and dCAF-82 reactivity towards ^•^OOH increases with the deprotonation degree. In water, considering the pH = 7.4, the global rate coefficients are diffusion limited and around the order of 10^9^ M^−1^ s^−1^.

**Table 4:** Energy barriers (Δ*G*^≠^, kcal/mol) for the reactions of dCAF-2, dCAF-16, and dCAF-82 with ^●^OOH, in water (W) and pentylethanoate (PE), at 298.15 K

	PE		Water	
	Neutral	Neutral	Mono-anion	Di-anion
dCAF-2				
*f*-HAT				
*O7a*	8.10	15.20	14.12	na
*O8a*	17.77	15.72	15.39	Barrier-less
dCAF-16				
*f*-HAT				
*O7a*	16.99	16.80	14.54	na
*O8a*	15.80	14.85	6.86	Barrier-less
dCAF-82				
*f*-HAT				
*O7a*	12.41	14.04	13.96	na
*O8a*	13.81	11.45	10.11	Barrier-less

*na* Non-available

**Table 5:** Rate constants (k, M^−1^ s^−1^) for the reactions of dCAF-2, dCAF-16, and dCAF-82 with ^●^OOH, in water (W) and pentylethanoate (PE), at 298.15 K

	PE		Water	
	Neutral	Neutral	Mono-anion	Di-anion
dCAF-2				
*f*-HAT				
*O7a*	3.45 × 10^3^	1.08 × 10^4^	3.58 × 10^4^	na
*O8a*	1.98 × 10^2^	8.88 × 10^3^	5.30 × 10^3^	7.86 × 10^9^
*k*_Total_	3.65 × 10^3^	1.97 × 10^4^	4.11 × 10^4^	7.86 × 10^9^
*k*_Global_			1.76 × 10^9^	
dCAF-16				
*f*-HAT				
*O7a*	1.81 × 10^3^	2.98 × 10^3^	5.08 × 10^4^	na
*O8a*	1.92 × 10^3^	1.96 × 10^4^	6.77 × 10^4^	8.03 × 10^9^
*k*_Total_	3.73 × 10^3^	2.26 × 10^4^	1.18 × 10^5^	8.03 × 10^9^
*k*_Global_			3.19 × 10^9^	
dCAF-82				
*f*-HAT				
*O7a*	8.04 × 10^4^	1.69 × 10^5^	9.39 × 10^4^	na
*O8a*	5.87 × 10^3^	4.43 × 10^5^	2.97 × 10^6^	7.98 × 10^9^
*k*_Total_	8.63 × 10^4^	6.12 × 10^5^	3.06 × 10^6^	7.98 × 10^9^
*k*_Global_			1.79 × 10^9^	

*na* Non-available

In pentylethanoate solution, the total rate constant for the dCAF-82 + ^•^OOH reaction was estimated to be 8.63 × 10^4^ M^−1^ s^−1^, which indicates that this derivative is 2.2 times faster than caffeic acid (3.93 × 10^4^ M^−1^ s^−1^, [1]) for scavenging this kind of free radicals. However, in the case of dCAF-2 and dCAF-16, their total rate constants in lipid medium are lower than the caffeic acid, at least in one order of magnitude. Moreover, considering that lipidic peroxidation of the cell’s membranes occurs at 1.18 × 10^3^ M^−1^ s^−1^ [58], all designed dCAF and its parent molecule are likely to provide chemical protection against such a deleterious process and, consequently, to act as inhibitors of the associated cellular damage.

In aqueous solution, at pH = 7.4, the difference is even larger. The global rate coefficients for the dCAF-2, dCAF-16, and dCAF-82 + ^•^OOH reactions are 1.76 × 10^9^ M^−1^ s^−1^, 3.19 × 10^9^ M^−1^ s^−1^, and 1.79 × 10^9^ M^−1^ s^−1^, respectively, while that of caffeic acid under similar conditions is 2.69 × 10^8^ M^−1^ s^−1^, i.e., the reactions of designed dCAF are at least five times faster than that of the parent molecule. Based on the obtained kinetic data, the designed dCAF is also predicted to be more efficient than Trolox, which is considered a reference antioxidant (8.96 × 10^4^ M^−1^ s^−1^, [59, 60]), and caffeic acid metabolites such as ferulic acid (3.36 × 10^8^ M^−1^ s^−1^, [1]), *p*-coumaric acid (8.51 × 10^7^ M^−1^ s^−1^, [1]), and dihydrocaffeic acid (1.04 × 10^8^ M^−1^ s^−1^, [1]).

### The reaction force

**Fig. 4:**
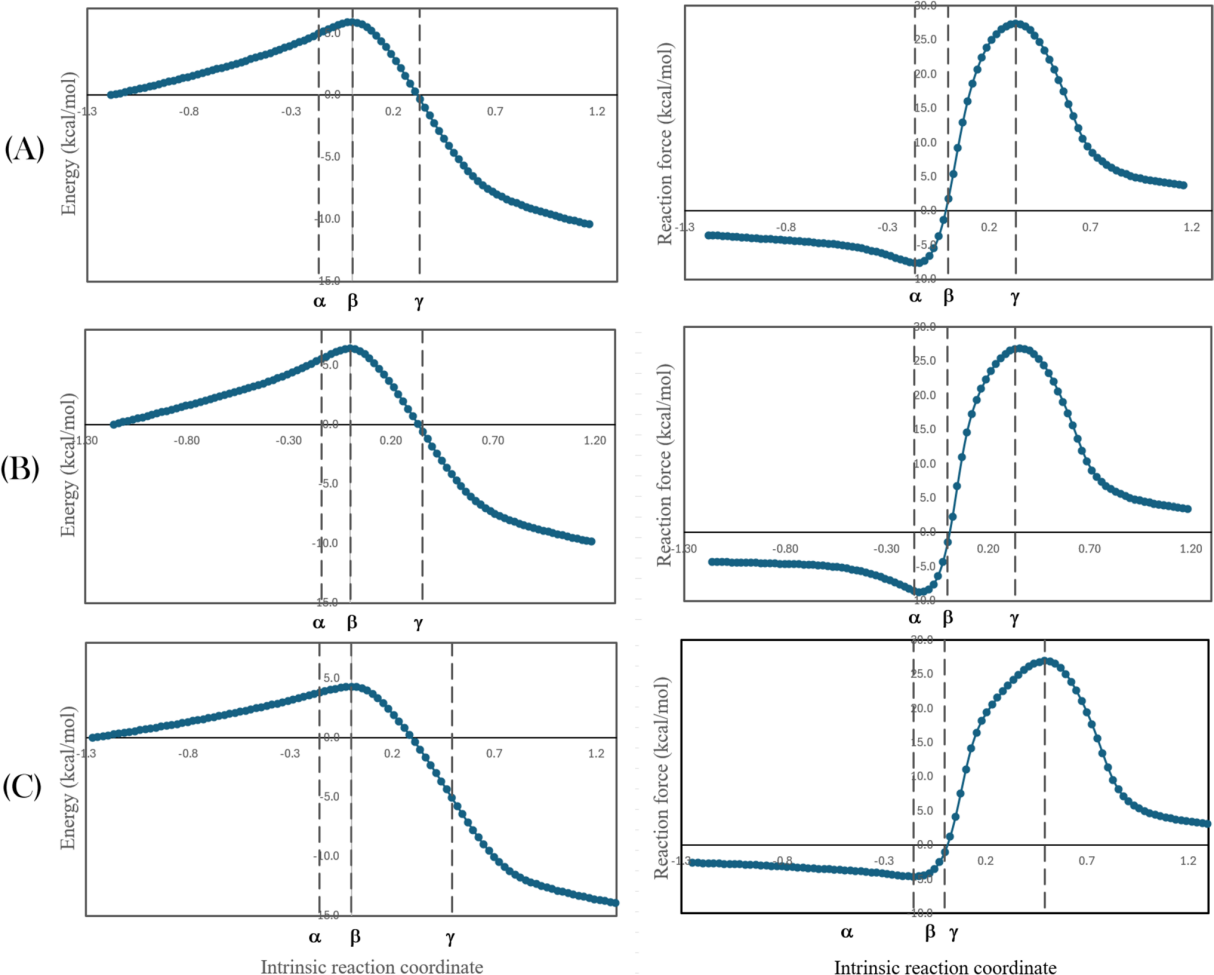
Energy and reaction force along intrinsic reaction coordinate for the most important reaction channel of the reactions between dCAF-2 (**A**), dCAF-15 (**B**), and dCAF-82 (**C**) anions with the OOH radical

The reaction force (RF) model allows analyzing the potential energy profile of chemical reactions using the negative of its derivative with respect to the reaction coordinate. It is calculated as the negative of the potential energy derivative with respect to the reaction coordinate. The RF provides details on the physical phenomena involved in the energy variations that go along such chemical reactions [61]. The potential energy V(IRC) and the reaction force RF(IRC) along intrinsic reaction coordinate (IRC), are presented in Fig. 4 for the most important reaction channel of the reactions between dCAF-2, dCAF-16, and dCAF-82 anions with the OOH radical. Three points (α, β, and γ) define four zones in these figures [46]. They are the following:

- First zone (from reactants to IRC = *α*): characterized by a retarding force that leads to a minimum at IRC = *α*. This zone has been associated with preparation, including conformational changes and rotations, required to facilitate the reaction, probably by bringing closer, and reorienting, the interacting sites.
- Second zone (from IRC = *α* to IRC = *β*): a driving force appears and starts counteracting the retarding one, although the overall effect remains retarding, up to IRC = *β* where both forces are equal, i.e., RF(IRC) = 0, that corresponds to the transition state (TS). Thus, in terms of RF, the TS is at equilibrium, despite being a maximum in energy terms. In this zone, the transition from reactants to products (breaking and forming bonds) starts to be significant.
- Third zone (from IRC = *β* to IRC = *γ*): the driving force dominates and riches its maximum at IRC = *γ*. In this zone, the transition from reactants to products (breaking and forming bonds) finishes to be significant.
- Fourth zone (from IRC = *γ* to products): the driving force weakens until become zero for the products. In this zone, the system relaxes until the equilibrium geometry of the products is reached.

Accordingly, the activation energy (Δ*E*^≠^) can be seen as the result of two contributions, i.e., the retarding and the driving forces. Their relative weight can be qualitatively estimated by comparing the areas under the RF(IRC) curve for the first and second zones.

Some relevant energies are reported in Table 6. According to them, the O atom in the OOH radical is a better H acceptor than the phenolic OH of anionic derivatives. Such a trend promotes the O–H interactions in the products to a larger extent than in the reactants. Therefore, the reaction force analysis predicts that Δ*E*^≠^ is higher for the reverse process than for the forward reaction. The Δ*E*^≠^ decomposing into two components justifies this based on the energy associated to the preparation step, which is larger for products → γ than for reactants → *α* (Table 6). This difference was found to be equal to 5.20, 7.74, and 5.09 kcal/mol for dCAF-2, dCaf-16, and dCaf-82, respectively. In addition, the Δ*E*^≠^ portion related to the *H* transfer is lower for the forward reaction, Δ*E* (*α* → *β*), than for the reverse process, Δ*E* (*γ* → *β*), by 5.29, 6.10, and 8.85 kcal/mol for dCAF-2, dCaf-16, and dCaf-82, respectively.

**Table 6:** Computed energies (M05-2X/6–311 + g(d,p) along the intrinsic reaction coordinate

	Δ*E* (kcal/mol)		
	dCAF-2	dCAF-16	dCAF-82
Δ*E*^≠^ forward	5.89	6.40	4.31
Δ*E* (reactants → *α*)	4.95	5.53	3.79
Δ*E* (*α* → *β*)	0.94	0.87	0.52
Δ*E*^≠^ reverse	16.38	16.24	18.24
Δ*E* (products → *γ*)	10.15	9.26	8.87
Δ*E* (*γ* → *β*)	6.22	6.98	9.37

## Conclusions

By incorporating different functional groups to the caffeic acid molecular framework, 3829 caffeic acid derivatives (dCAF) were designed, using the CADMA-Chem computational protocol. The generated chemical space was sampled using a selection score that accounts for ADME properties, toxicity, and synthetic accessibility. This score allowed to identify 645 candidates that surpass, in this context, the parent molecule and a reference set of molecules currently in use as neuroprotectors. For the best three, BDE and IE reactivity descriptors were calculated and compared with those of caffeic acid and Trolox. Based on such a comparison, the best candidates were selected (dCAF-2, dCAF-16, and dCAF-82).

A comprehensive investigation of the antioxidant behavior of these compounds were then performed at the M05-2X/6–311 + G(d,p) level of theory. To explore their radical scavenging activity, the thermochemistry and kinetics of the dCAF-2, dCAF-16, and dCAF-82 + ^•^OOH reactions were evaluated considering environments of different polarity and three different reaction mechanisms, namely single electron transfer, formal hydrogen transfer, and radical adduct formation. According to the gathered data, designed dCAF are predicted to be more efficient as a peroxyl radical scavenger than the parent molecule, Trolox, and several chemical compounds regarded as excellent antioxidants.

The reaction path contributing the most to this behavior, for the most abundant acid–base species of dCAF-2, dCAF-16, and dCAF-82 in aqueous solution, at physiological pH, was analyzed in terms of the reaction force. Such an analysis supported that the O atom in the OOH radical is a better H acceptor than the O in the phenol group of anionic dCAF-82. Thus, the activation energy is higher for the reverse process than for the forward reaction, which can also be justified based on the energy associated to the preparation step.

## Supplementary Information

Below is the link to the electronic supplementary material.

Supplementary file1 (PDF 3028 KB)

## Data Availability

No datasets were generated or analysed during the current study.
