# Supplementary material for: Computer-aided design of caffeic acid derivatives: free radical scavenging activity and reaction force
Source: J Mol Model. 2024 Dec 27;31(1):30. doi: 10.1007/s00894-024-06226-2 (PMC11680653; doi:10.1007/s00894-024-06226-2)
Supplement: Supplementary file 1 — Supplementary file1 (PDF 3028 KB) [file 894_2024_6226_MOESM1_ESM.pdf]

Computer-aided Design of Caffeic Acid Derivatives: Free Radical scavenging activity and Reaction Force

B. Carolina Morales-García,[1] Adriana Pérez-González,[2] J. Raúl Álvarez-Idaboy[3] and Annia Galano,[1*]

[1] Departamento de Química. Universidad Autónoma Metropolitana-Iztapalapa. Avenida Ferrocarril San Rafael Atlixco, número 186, Colonia Leyes de Reforma 1A Sección, Alcaldía Iztapalapa, Código Postal 09310, Ciudad de México, México.

[2] CONAHCYT - Universidad Autónoma Metropolitana - Iztapalapa. Departamento de Química. Avenida Ferrocarril San Rafael Atlixco, número 186, Colonia Leyes de Reforma 1A Sección, Alcaldía Iztapalapa, Código Postal 09310, Ciudad de México, México.

[3] Universidad Nacional Autónoma de México, Facultad de Química, Departamento de Física y Química Teórica, Ciudad de México, Código Postal 04510, México.

Table of Contents

Scheme S1. Deprotonation routes for the designed caffeic acid derivatives chosen as the most promising, from their drug-like behavior. 3

Fig. S1. Selection score (S^S) for the designed caffeic acid derivatives in this work. Vertical lines mark the arithmetic mean of the reference set (red) and the score for parent molecule (caffeic acid, green). 4

Fig. S2. Species distribution diagram at different *p*H, for the designed caffeic acid derivatives chosen as the most promising, from their drug-like behavior. The vertical line landmarks the physiological *p*H (*p*H=7.4). 5

Fig.S3. Transition states of the *f*-HAT mechanism for the N: neutral and A: mono-anion species of the dCAF-2, in W:Water and PE:Pentylethanoate. 6

Fig.S4. Transition states of the *f*-HAT mechanism for the N: neutral and A: mono-anion species of the dCAF-16, in W:Water and PE:Pentylethanoate 7

Fig.S5. Transition states of the *f*-HAT mechanism for the N: neutral and A: mono-anion species of the dCAF-82, in W:Water and PE:Pentylethanoate 8

Fig. S6. Scan coordinate of the *f*-HAT mechanism from the O7a site for the di-anion of dCAF-2. (A) Products → Reactives. (B) Reactives → Products. 9

Fig. S7. Scan coordinate of the *f*-HAT mechanism from the O7a site for the di-anion of dCAF-16. (A) Products → Reactives. (B) Reactives → Products. 10

Fig. S8. Scan coordinate of the *f*-HAT mechanism from the O8a site for the di-anion of dCAF-82. (A) Products → Reactives. (B) Reactives → Products. 11

* E-mail: agalano@prodigy.net.mx, agal@xanum.uam.mx

Table S1. ΔE (kcal/mol) for the different conformational structures for dCAF-2, dCAF-16 and dCAF-82. 12

Table S2. Reference set of molecules, used to compare the estimated properties of caffeic acid derivatives. 15

Table S3. Estimated ADME properties for the reference set of pharmaceutical drugs for neurodegenerative diseases. Octanol/water partition coefficient (logP), polar surface area (PSA), number of non-hydrogen atoms (AtX), molecular weight (MW), number of acceptors in H-bridge interactions (HBA), number of donors in H-bridge interactions (HBD), number of rotatable bonds (RB), molar refractivity (MR). Estimated toxicity, expressed as LD_{50} and Ames mutagenicity (M); and synthetic accessibility (SA). 21

Selection Score (S^S) and elimination scores (S^E) 24

Table S4. Values of the ADME properties, toxicity and synthetic accessibility for the twenty designed caffeic acid derivatives (dCAF) with higher S^S. Oral rat 50 percent lethal dose (LD_{50}), Ames mutagenicity (M) and synthetic accessibility (SA). log P, polar surface area (PSA), number of non-hydrogen atoms (AtX), molecular weight (MW), number of acceptors in H-bridge interactions (HBA), number of donors in H-bridge interactions (HBD), number of rotatable bonds (RB), molar refractivity (MR). Also the S^S is presented. 26

Table S5. Elimination scores (S^E) for the subset of twenty designed caffeic acid derivatives (dCAF) chosen as the most promising, according to S^S. 27

Table S6. Reactivity indexes, their acronyms, calculation method and interpretation. 28

Table S7. First ionization energy (IE, eV) and the lowest bond dissociation energies (*l*-BDE, kcal/mol) for the designed caffeic acid derivatives (dCAF) chosen as the most promising, according to S^S. 29

Table S8. Tunneling values for the reactions of dCAF-2, dCAF-16 y dCAF-82 with •OOH, in water (W) and pentylethanoate (PE), at 298.15 K. 30

dCAF-2

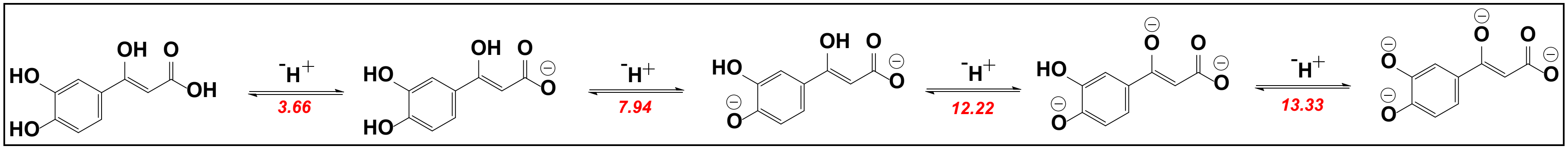

dCAF-16

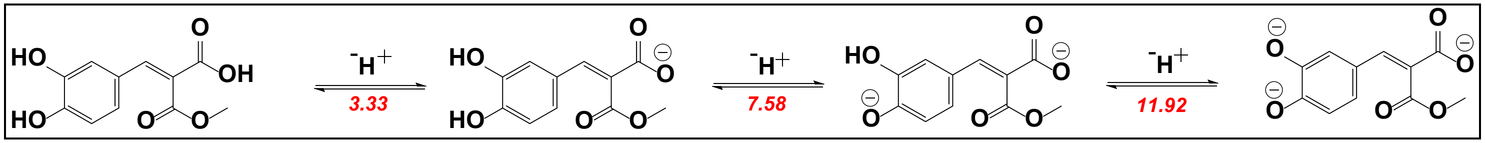

dCAF-82

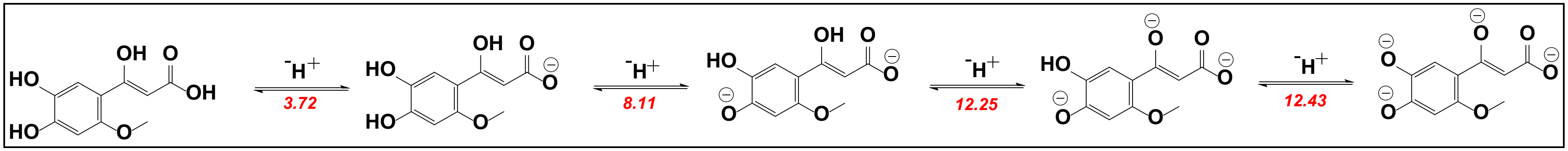

Scheme S1. Deprotonation routes for the designed caffeic acid derivatives chosen as the most promising, from their drug-like behavior.

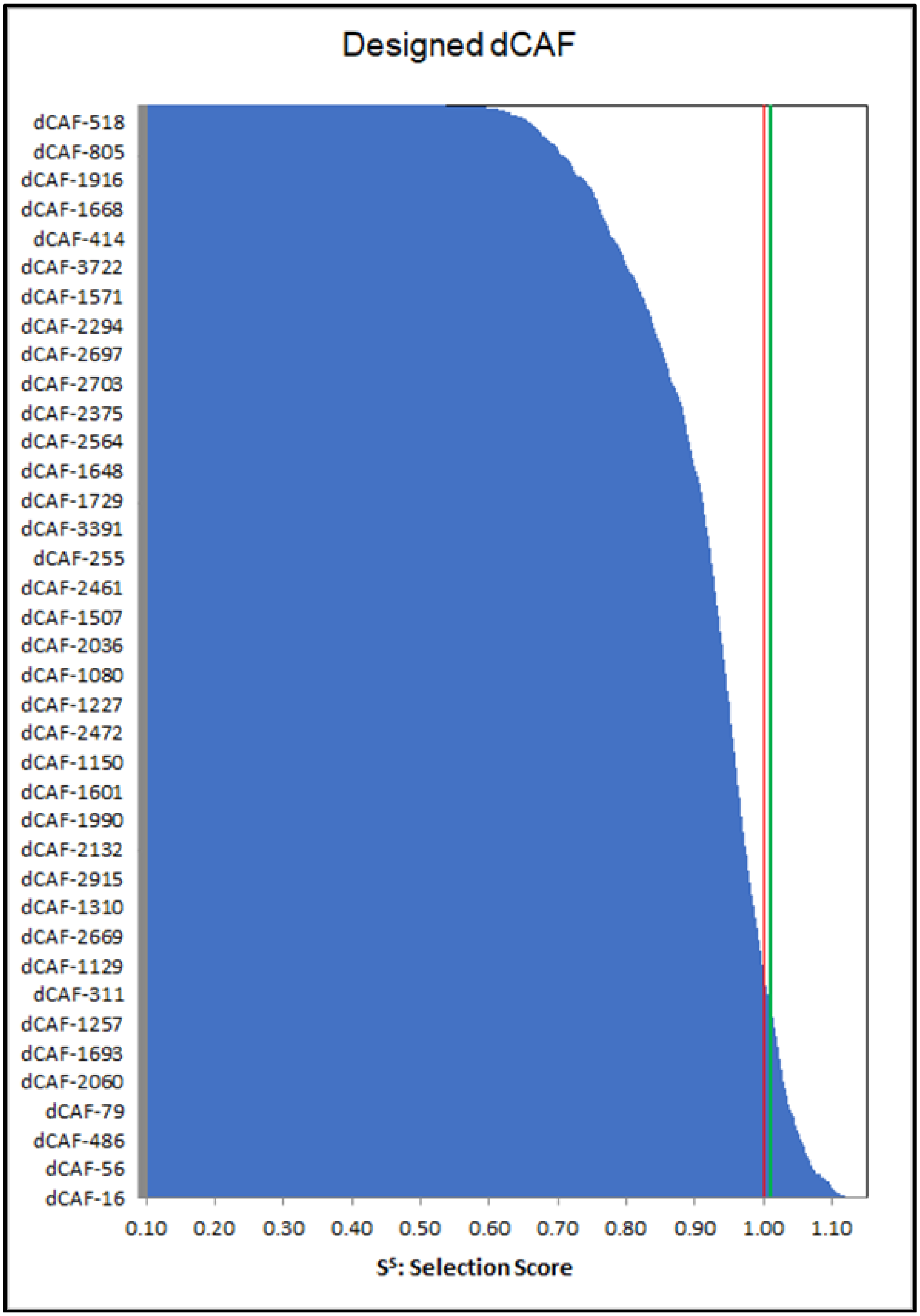

Fig. S1. Selection score (S^S) for the designed caffeic acid derivatives in this work. Vertical lines mark the arithmetic mean of the reference set (red) and the score for parent molecule (caffeic acid, green).

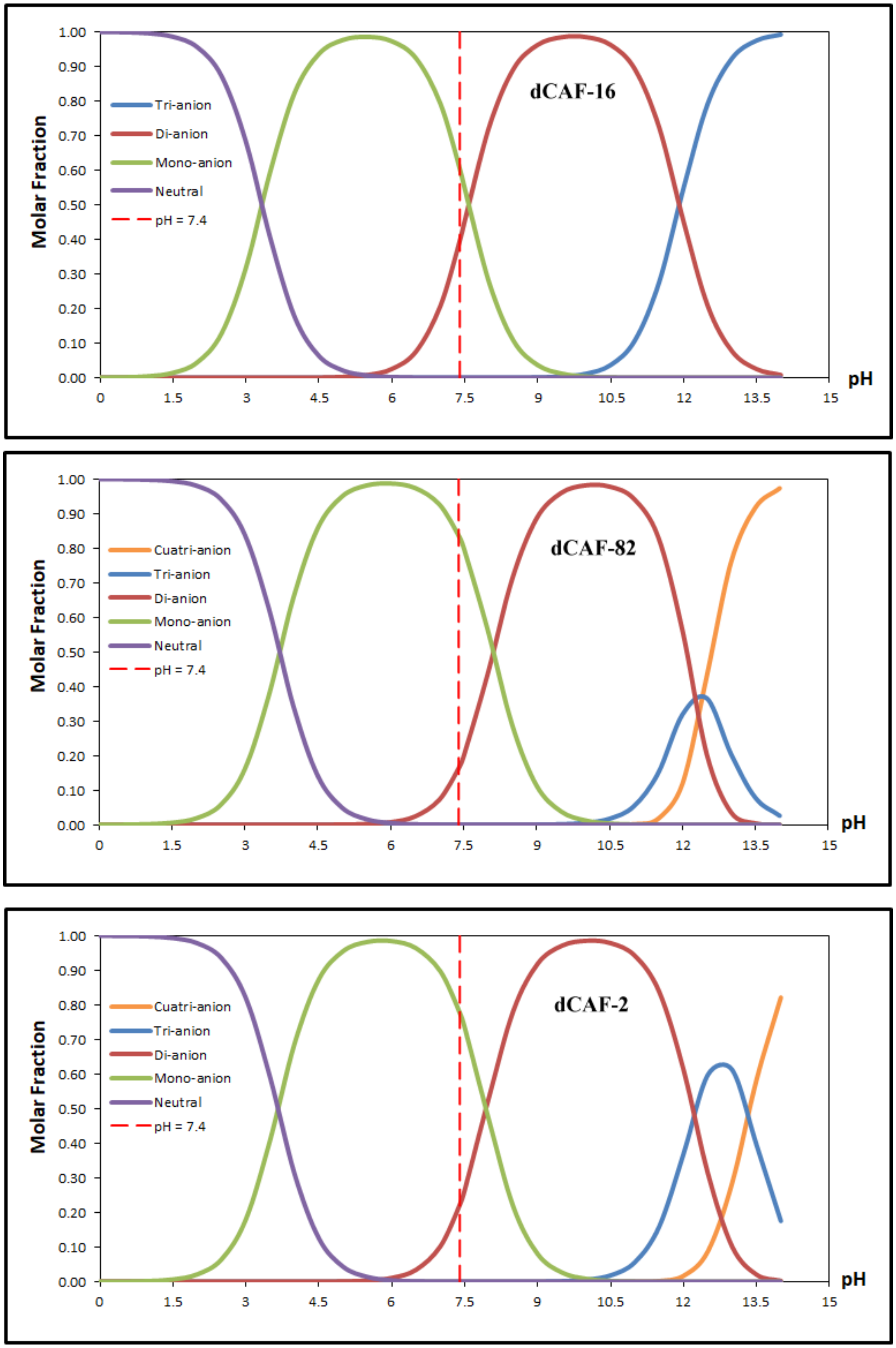

Fig. S2. Species distribution diagram at different *p*H, for the designed caffeic acid derivatives chosen as the most promising, from their drug-like behavior. The vertical line landmarks the physiological *p*H (*p*H=7.4).

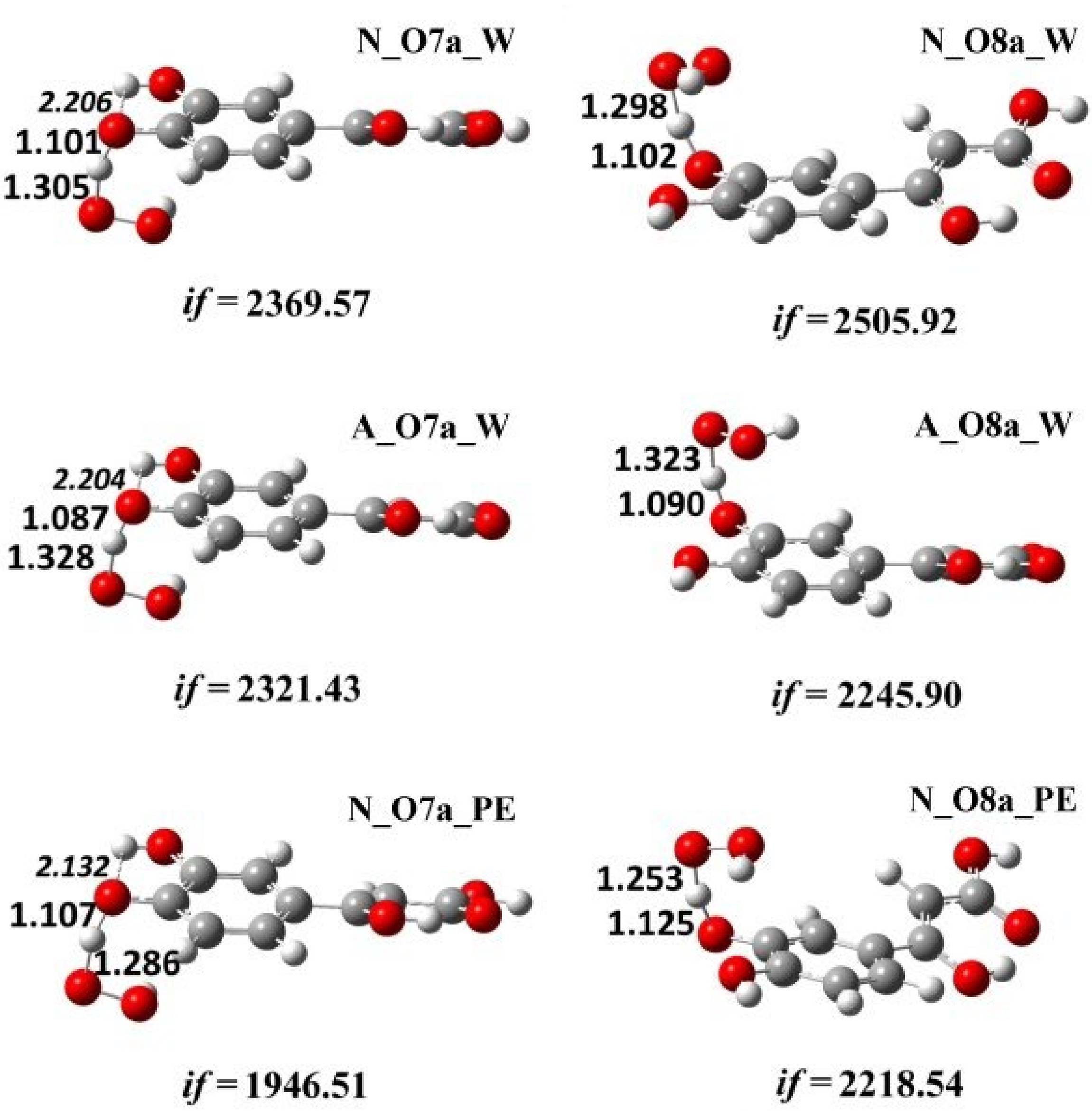

Fig.S3. Transition states of the *f*-HAT mechanism for the N: neutral and A: mono-anion species of the dCAF-2, in W:Water and PE:Pentylethanoate.

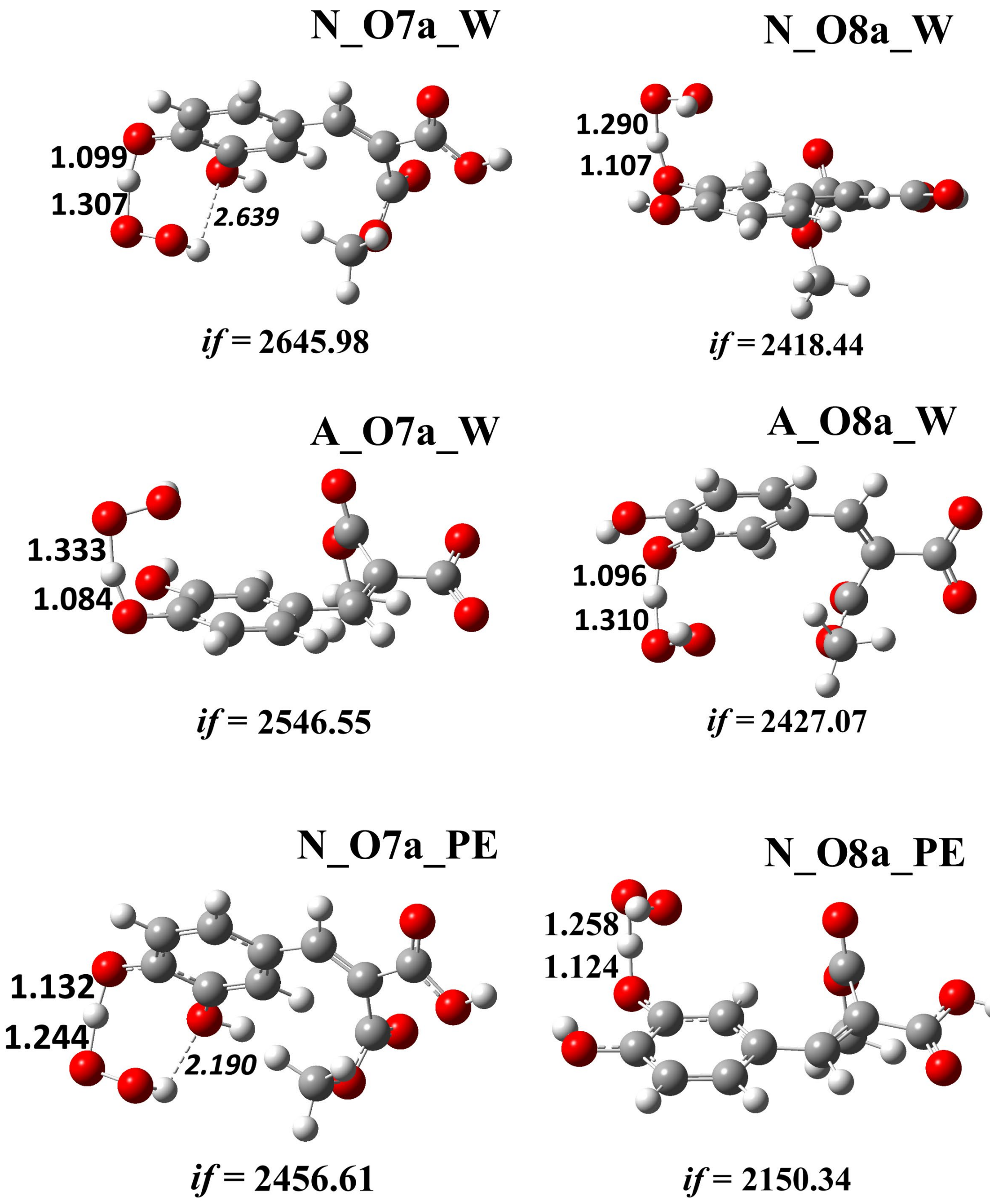

Fig.S4. Transition states of the *f*-HAT mechanism for the N: neutral and A: mono-anion species of the dCAF-16, in W:Water and PE:Pentylethanoate.

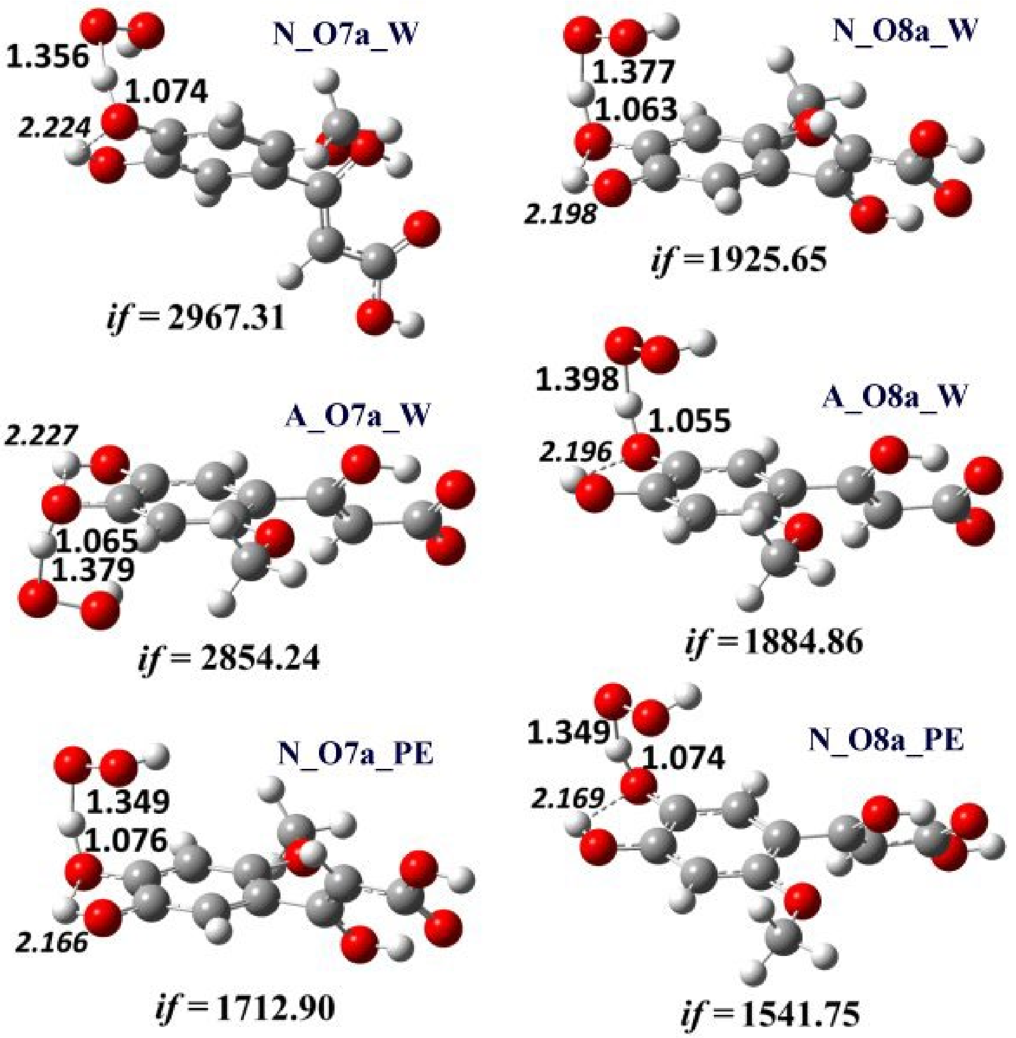

Fig.S5. Transition states of the *f*-HAT mechanism for the N: neutral and A: mono-anion species of the dCAF-82, in W:Water and PE:Pentylethanoate.

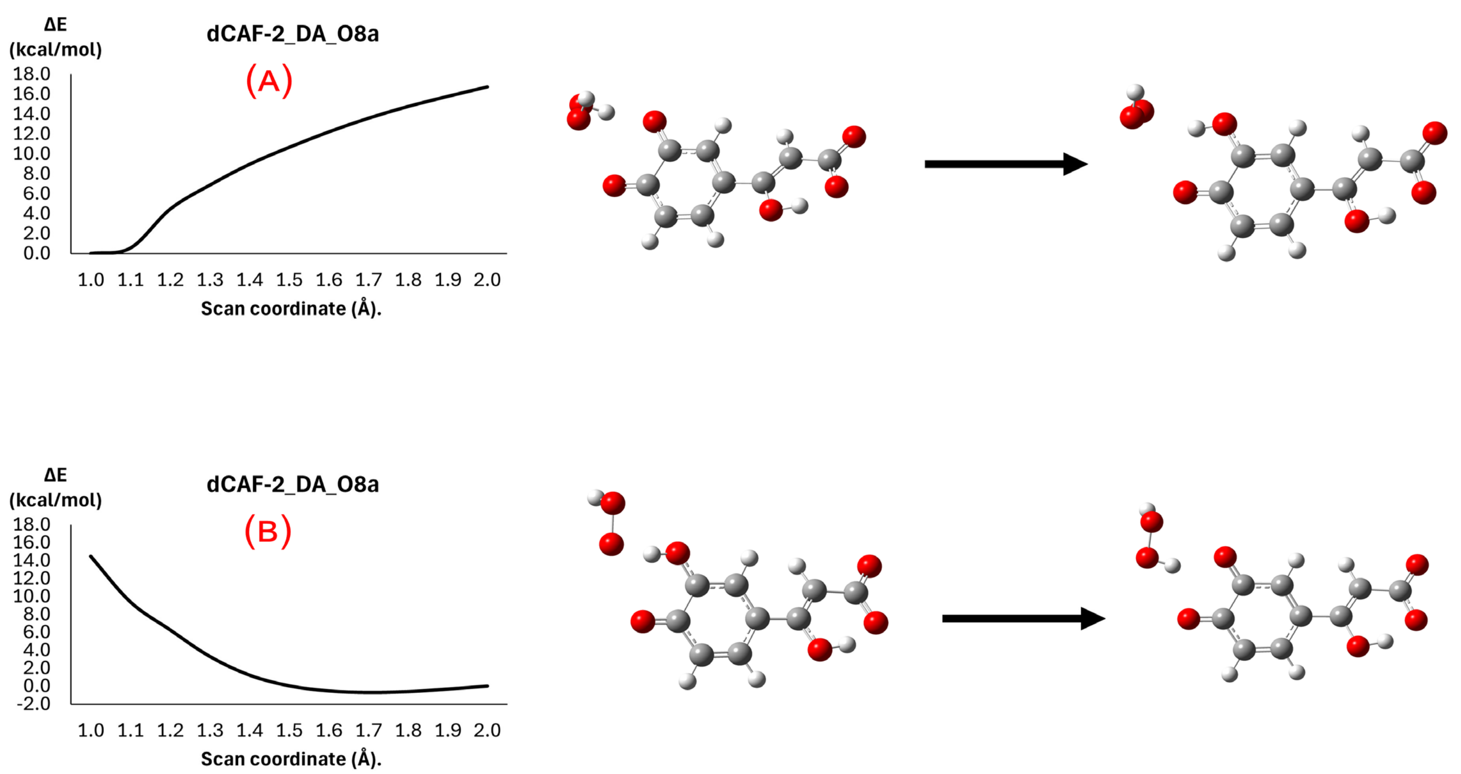

Fig. S6. Scan coordinate of the *f*-HAT mechanism from the O7a site for the di-anion of dCAF-2. (A) Products → Reactives. (B) Reactives → Products.

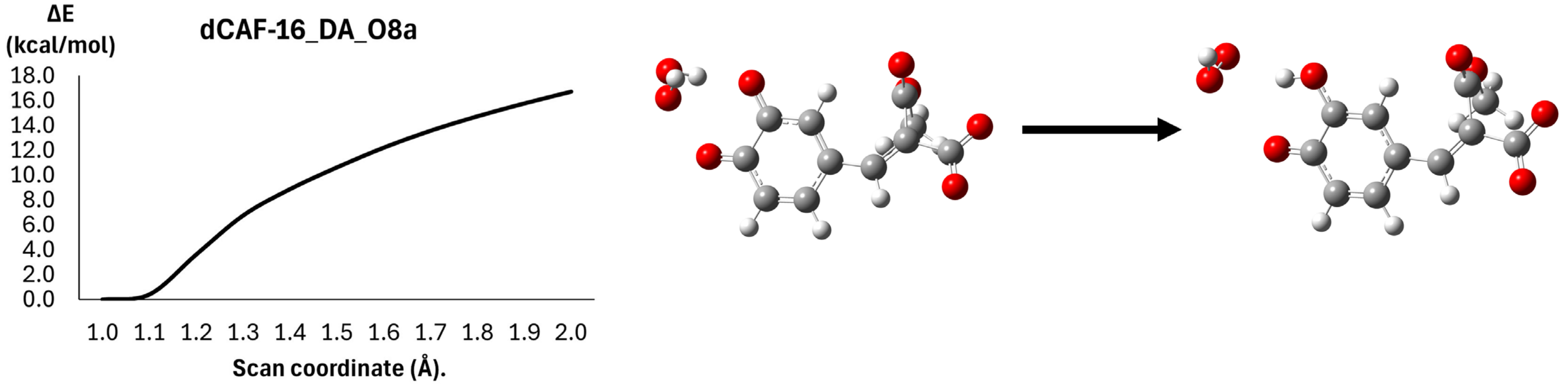

Fig. S7. Scan coordinate of the *f*-HAT mechanism from the O7a site for the di-anion of dCAF-16. (A) Products → Reactives. (B) Reactives → Products.

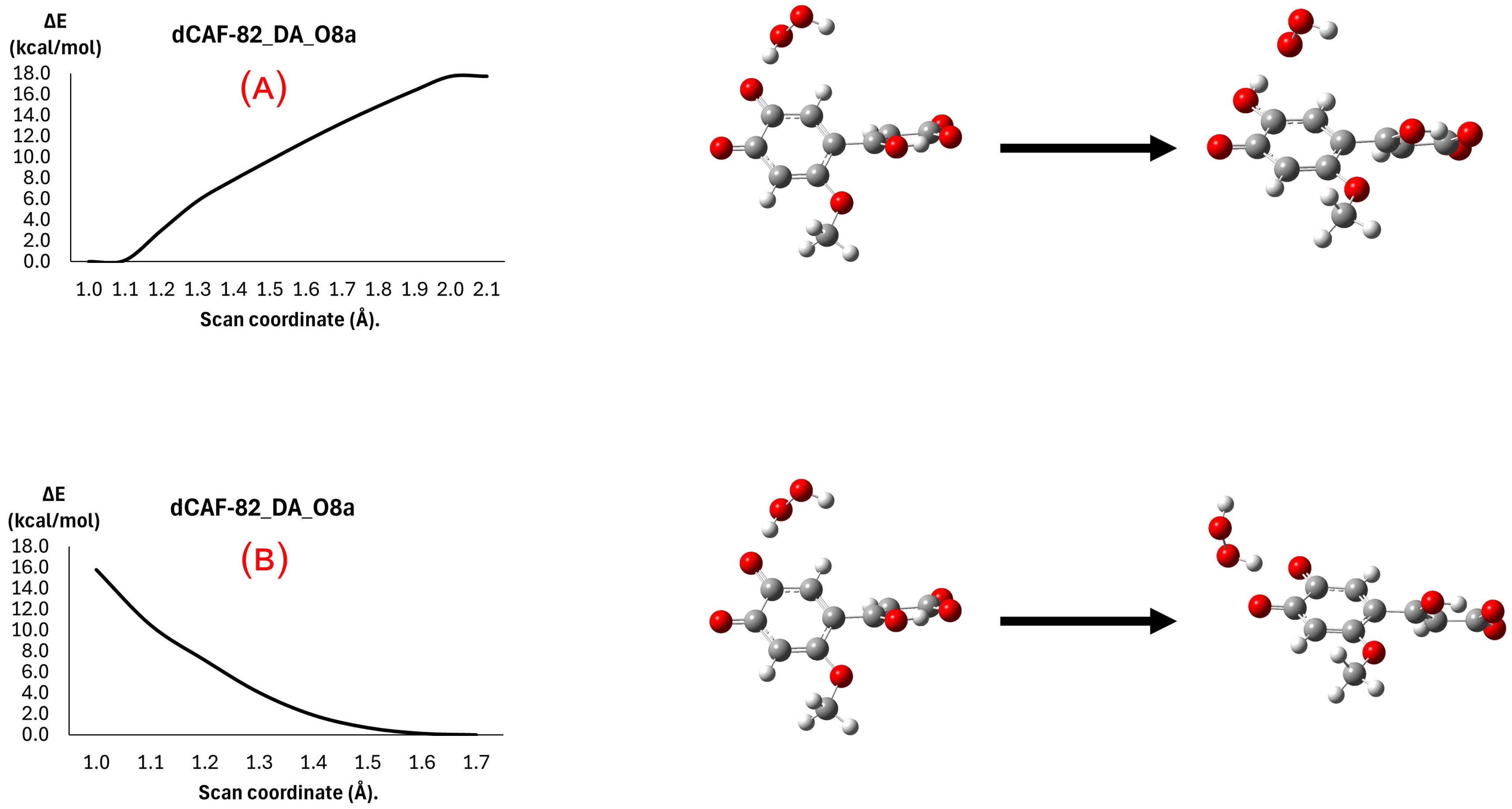

Fig. S8. Scan coordinate of the *f*-HAT mechanism from the O8a site for the di-anion of dCAF-82. (A) Products → Reactives. (B) Reactives → Products.

Table S1. ΔE (kcal/mol) for the different conformational structures for dCAF-2, dCAF-16 and dCAF-82.

Conformer number	dCAF-2	Rotatable dihedral angle	ΔE(kcal/mol)
	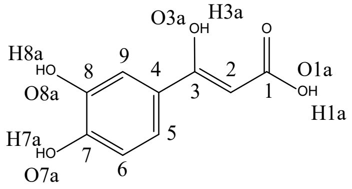		
1	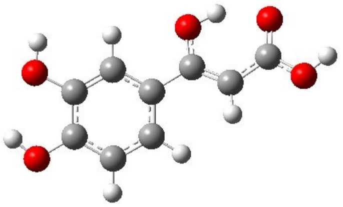	D1(6,7,O7a,H7a)= -178.60333<br>D2(9,8,O8a,H8a)= 1.96093<br>D3(9,4,3,O3a)= -18.74750<br>D4(4,3,O3a,H3a)= 178.76954<br>D5(3,2,1,O1a)= -178.78485	0.00
2	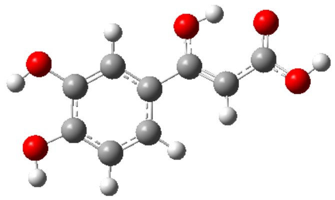	D1(6,7,O7a,H7a)= -1.54785<br>D2(9,8,O8a,H8a)= -179.49453<br>D3(9,4,3,O3a)= -19.29136<br>D4(4,3,O3a,H3a)= 178.82678<br>D5(3,2,1,O1a)= -178.72168	0.13
3	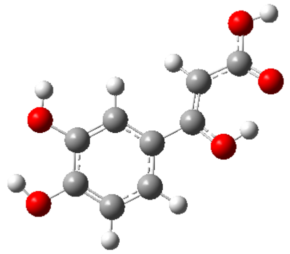	D1(6,7,O7a,H7a)= -179.86585<br>D2(9,8,O8a,H8a)= 0.83175<br>D3(9,4,3,O3a)= 161.81446<br>D4(4,3,O3a,H3a)= 178.71900<br>D5(3,2,1,O1a)= 179.33390	0.17
4	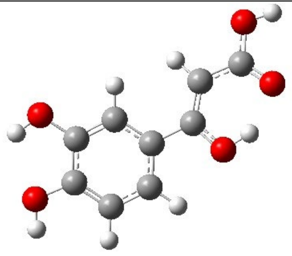	D1(6,7,O7a,H7a)= 0.80276<br>D2(9,8,O8a,H8a)= 179.96566<br>D3(9,4,3,O3a)= 162.48538<br>D4(4,3,O3a,H3a)= 178.86506<br>D5(3,2,1,O1a)= 179.36612	0.12

Conformer number	dCAF-16	Rotatable dihedral angle	ΔE(kcal/mol)
	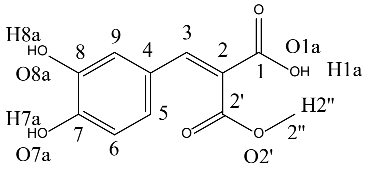		
1	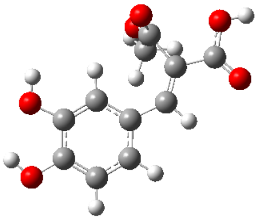	D1(6,7,O7a,H7a)= 179.21353<br>D2(9,8,O8a,H8a)= -0.45256<br>D3(3,2,1,O1a)= 179.26109	0.00
2	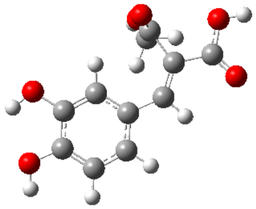	D1(6,7,O7a,H7a)= 1.76844<br>D2(9,8,O8a,H8a)= 179.96402<br>D3(3,2,1,O1a)= 179.61720	0.11
3	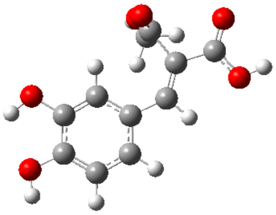	D1(6,7,O7a,H7a)= 1.93996<br>D2(9,8,O8a,H8a)= 179.67166<br>D3(3,2,1,O1a)= -2.62372	0.33
Conformer number	dCAF-82	Rotatable dihedral angle	ΔE(kcal/mol)
	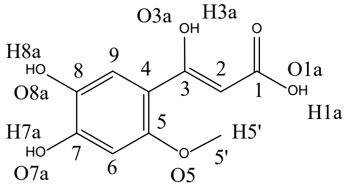		
1	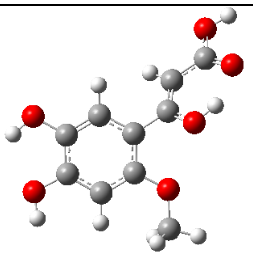	D1(6,7,O7a,H7a)= 1.42856<br>D2(9,8,O8a,H8a)= -179.04008<br>D3(6,5,O5,5’)= 1.55923<br>D4(5,4,3,O3a)= -49.08172<br>D5(3,2,1,O1a)= -179.47262	0.00

2	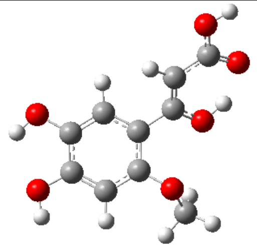	D1(6,7,O7a,H7a)= -4.71376<br>D2(9,8,O8a,H8a)= -179.45639<br>D3(6,5,O5,5')= 66.48289<br>D4(5,4,3,O3a)= 45.25018<br>D5(3,2,1,O1a)= 178.84558	1.47
3	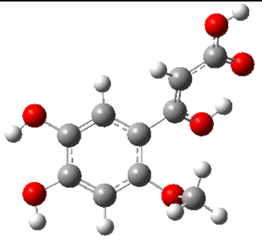	D1(6,7,O7a,H7a)= -0.48768<br>D2(9,8,O8a,H8a)= 178.04267<br>D3(6,5,O5,5')= -101.56109<br>D4(5,4,3,O3a)= 41.08451<br>D5(3,2,1,O1a)= 179.74795	1.65

Table S2. Reference set of molecules, used to compare the estimated properties of caffeic acid derivatives.

Compound (CAS)	Canonical Smile	Structure	Compound (CAS)	Canonical Smile	Structure
Acetylcarnitine (3040-38-8)	[O-]C(CC(C[N+](C)(C)C)OC(C)=O)=O	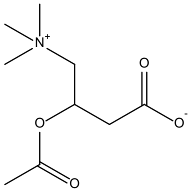	Levitiracetam (102767-28-2)	O=C1CCCN1[C@@H](CC)C(N)=O	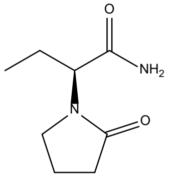
Amantadine (768-94-5)	NC12CC3CC(C2)CC(C1)C3	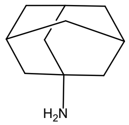	Lisuride (18016-80-3)	[H][C@@]12CC3=CNC4=CC=CC(C1=C[C@H](NC(N(CC)CC)=O)CN2C)=C34	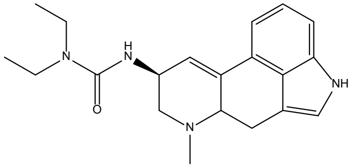
Apomorphine (58-00-4)	CN1CCC2=CC=CC3=C2C1CC4=C3C(O)=C(O)C=C4	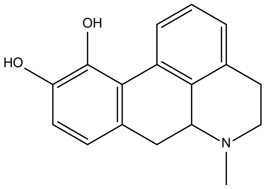	Masitinib (790299-79-5)	CC1=C(NC2=NC(C3=CC=CN=C3)=CS2)C=C(NC(C4=CC=C(CN5CCN(C)CC5)C=C4)=O)C=C1	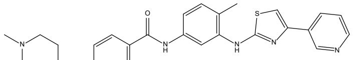
Atuzaginstat (2211981-76-7)	FC1=C(F)C=CC(F)=C1OCC([C@@H](NC(C2CCCC2)=O)CCCCN)=O	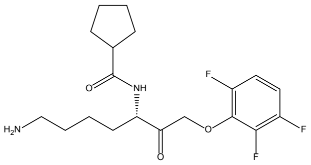	Melatonin (73-31-4)	O=C(C)NCCC1=CNC2=CC=C(C=C12)OC	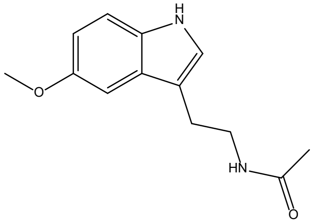

Baclofen (1134-47-0)	NCC(C1=CC=C(C=C1)Cl)CC(O)=O	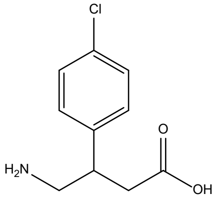	Memantine (19982-08-2)	CC12CC3CC(C1)(CC(C3)(C2)N)C	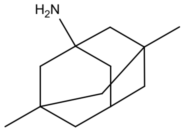
Benserazide (14919-77-8)	NC(C(NNCC1=CC=C(C(O)=C1O)O)=O)CO	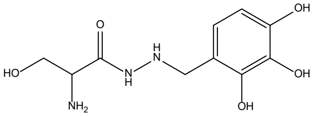	Metformin (1115-70-4)	CN(C(NC(N)=N)=N)C	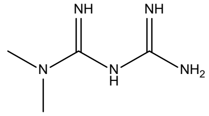
Benzatropine (86-13-5)	CN1C2CC(OC(C3=CC=CC=C3)C4=CC=CC=C4)CC1CC2	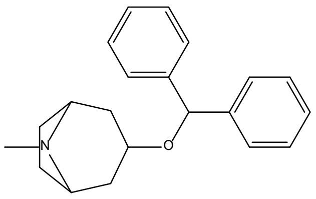	Modafinil (68693-11-8)	NC(CS(C(C1=CC=CC=C1)C2=CC=CC=C2)=O)=O	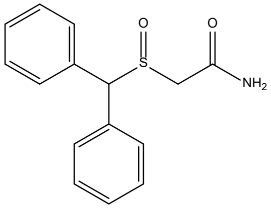
Biperiden (514-65-8)	OC(C1CC2CC1C=C2)(C3=CC=CC=C3)CCN4CCCCC4	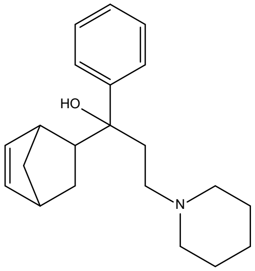	Nilotinib (641571-10-0)	CC1=CN(C=N1)C2=CC(NC(C3=CC(NC4=NC=CC(C5=CC=CN=C5)=N4)=C(C)C=C3)=O)=CC(C(F)(F)F)=C2	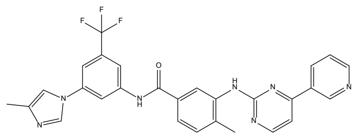
Blarcamesine (195615-83-9)	CN(C)CC1C(C2=CC=CC=C2)(C3=CC=CC=C3)OCC1	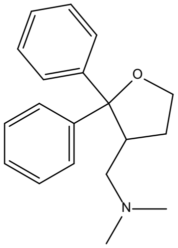	Opicapone (923287-50-7)	CC1=C(C(C)=[N+](C(Cl)=C1C2=NOC(C3=CC([N+]([O-])=O)=C(O)C(O)=C3)=N2)[O-])Cl	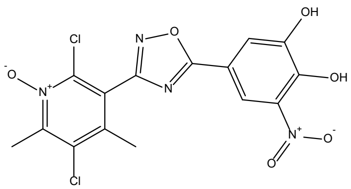

Brexpiprazole (913611-97-9)	O=C1NC2=CC(OCCCCN3CCN(C4=C5C=CSC5=CC=C4)CC3)=CC=C2C=C1	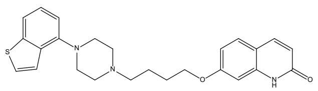	Pimavanserin (706779-91-1)	CC(C)COC1=CC=C(CNC(N(C2CCN(C)CC2)CC3=CC=C(F)C=C3)=O)C=C1	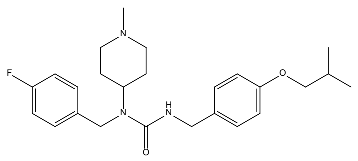
Bromocriptine (25614-03-3)	BrC1=C2C3=C(N1)C=CC=C3C4=C[C@@H](C(N[C@]5(C(N6[C@H](C(N7CCC[C@]7([C@@]6(O5)O)[H])=O)CC(C)C)=O)C(C)C)=O)CN(C)[C@@]([H])4C2	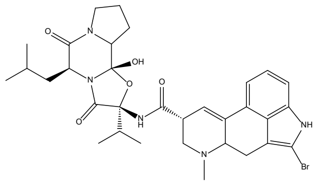	Piribedil (3605-01-4)	C1(OCOC1=C2)=CC=C2CN3CCN(C4=NC=CC=N4)CC3	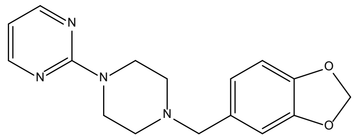
Cabergoline (81409-90-7)	[H][C@@]12CC3=CNC4=CC=CC([C@]1(C[C@@H](C(N(C(NCC)=O)CCCN(C)C)=O)CN2CC=C)[H])=C34	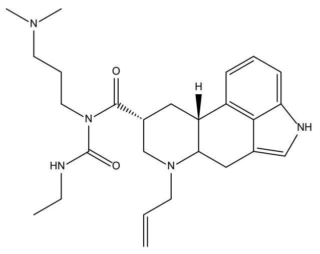	Pramipexole (104632-26-0)	CCCN[C@H]1CCC2=C(C1)SC(N)=N2	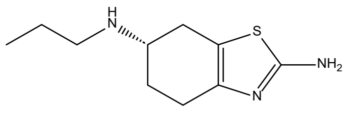
Caffeine (58-08-2)	O=C(N(C1=O)C)N(C2=C1N(C=N2)C)C	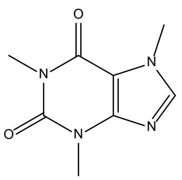	Procyclidine (77-37-2)	OC(C1CCCCC1)(CCN2CCCC2)C3=CC=CC=C3	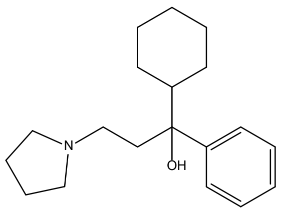

Carbidopa (28860-95-9)	C[C@@](NN)(C(O)=O)CC1=CC(O)=C(C=C1)O	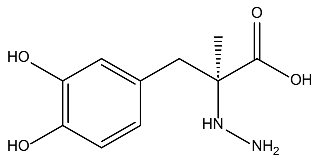	Rasagiline (136236-51-6)	C#CCN[C@@H]1CCC2=CC=CC=C12	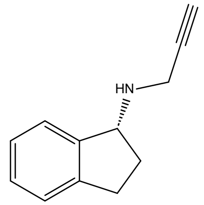
Curcumin (458-37-7)	O=C(/C=C/C1=CC=C(O)C(OC)=C1)CC(/C=C/C2=CC(OC)=C(O)C=C2)=O	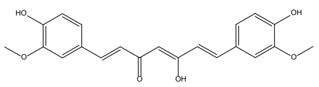	Remacemide (128298-28-2)	O=C(CN)NC(C1=CC=CC=C1)(C)CC2=CC=CC=C2	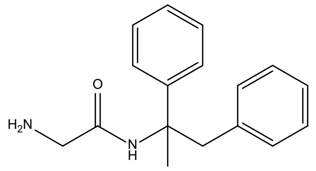
Dantrolene (7261-97-4)	O=C1NC(CN1/N=C/C2=CC=C(C3=CC=C(C=C3)[N+]([O-])=O)O2)=O	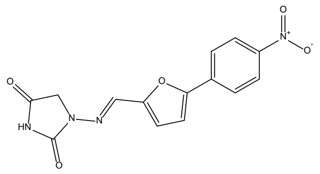	Riluzole (1744-22-5)	NC1=NC2=CC=C(OC(F)(F)F)C=C2S1	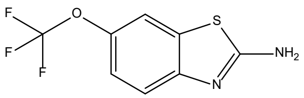
Donepezil (120014-06-4)	O=C1C(CC2=CC(OC)=C(OC)C=C21)CC3CCN(CC3)CC4=CC=CC=C4	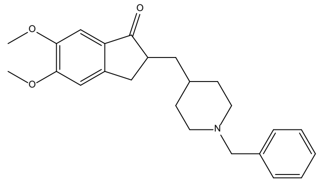	Rivastigmine (123441-03-2)	CCN(C(OC1=CC=CC([C@@H](N(C)C)C)=C1)=O)C	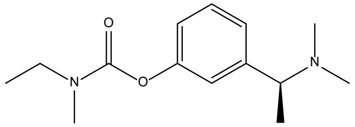
Eicosapentaenoic Acid (86227-47-6)	CCC=CCC=CCC=CCC=CCC=CCCCC(=O)OCC	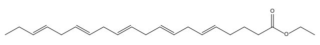	Ropinirole (91374-21-9)	O=C1NC2=C(C(CCN(CCC)CCC)=CC=C2)C1	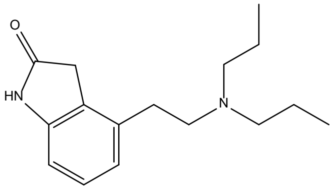

Entacapone (130929-57-6)	CCN(C(/C(C#N)=C/C1=CC([N+]([O-])=O)=C(C(O)=C1)O)=O)CC	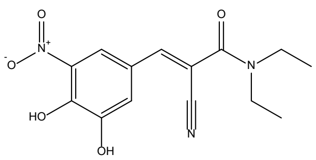	Rotigotine (99755-59-6)	CCCN([C@H]1CCC2=C(C=CC=C2C1)O)CCC3=CC=CS3	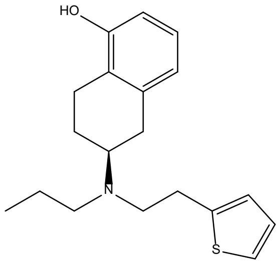
Escitalopram (128196-01-0)	FC(C=C1)=CC=C1[C@@]2(CCCN(C)C)C3=CC=C(C#N)C=C3CO2	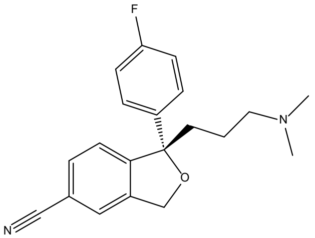	Selegiline (14611-51-9)	C[C@@H](N(C)CC#C)CC1=CC=CC=C1	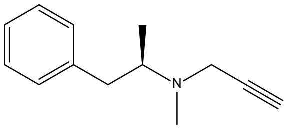
Fingolimod (162359-55-9)	NC(CO)(CO)CCC1=CC=C(CCCCCCCC)C=C1	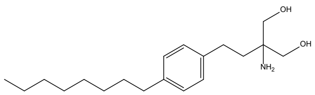	Simufilam (1224591-33-6)	CN1CCC2(NCC(N2CC3=CC=CC=C3)=O)CC1	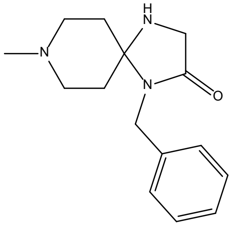
Galantamine (357-70-0)	[H][C@]12C[C@H](C=C[C@]13CCN(CC4=C3C(O2)=C(C=C4)OC)C)O	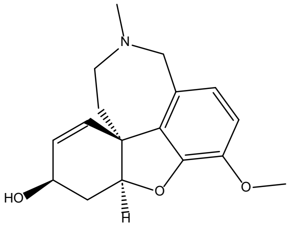	Tacrine (321-64-2)	NC1=C2C=CC=CC2=NC3=C1CCCC3	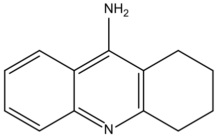

Guanfacine (29110-48-3)	NC(NC(CC1=C(Cl)C=CC=C1Cl)=O)=N	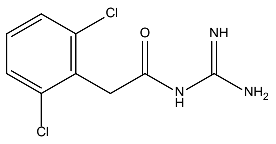	Tetrabenazine (58-46-8)	COC1=C(C=C2C3CC(C(CN3CCC2=C1)CC(C)C)=O)OC	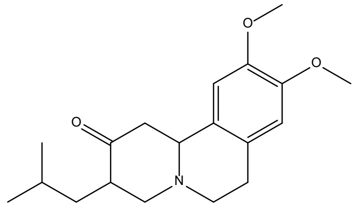
Hydralazine (86-54-4)	NNC1=NN=CC2=CC=CC=C12	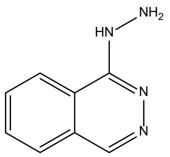	Tizanidine (51322-75-9)	ClC1=C(C2=NSN=C2C=C1)NC3=NCCN3	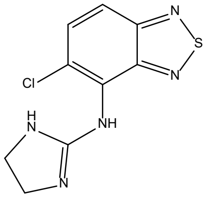
Istradefylline (155270-99-8)	COC1=C(OC)C=C(/C=C/C2=NC(N3CC)=C(N2C)C(N(CC)C3=O)=O)C=C1	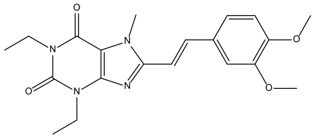	Tolcapone (134308-13-7)	OC1=C(O)C=C(C(C2=CC=C(C)C=C2)=O)C=C1[N+]([O-])=O	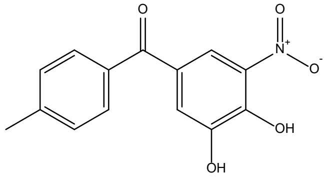
Ladostigil (209349-27-4)	C#CCN[C@H]1C2=CC(OC(N(C)CC)=O)=CC=C2CC1	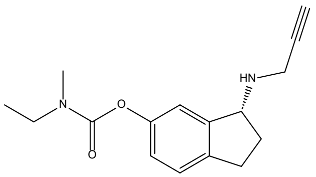	Trihexyphenidyl (144-11-6)	OC(C1=CC=CC=C1)(C2CCCCC2)CCN3CCCCC3	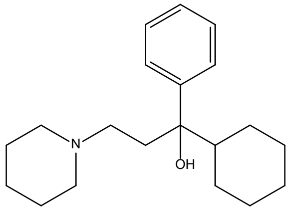
L-DOPA (59-92-7)	OC([C@@H](N)CC1=CC=C(O)C(O)=C1)=O	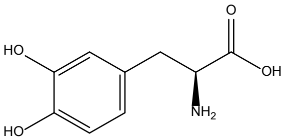	Valiltramiprosate (1034190-08-3)	CC(C)[C@H](N)C(NCCCS(=O)(O)=O)=O	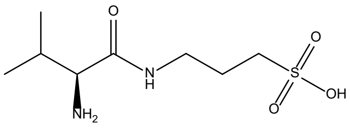

Table S3. Estimated ADME properties for the reference set of pharmaceutical drugs for neurodegenerative diseases. Octanol/water partition coefficient (logP), polar surface area (PSA), number of non-hydrogen atoms (AtX), molecular weight (MW), number of acceptors in H-bridge interactions (HBA), number of donors in H-bridge interactions (HBD), number of rotatable bonds (RB), molar refractivity (MR). Estimated toxicity, expressed as LD_{50} and Ames mutagenicity (M); and synthetic accessibility (SA).

	logP	PSA	AtX	MW	HBA	HBD	RB	MR	LD_{50}	M	SA
Acetylcarnitine	-1.24	66.43	14	203.24	4	0	5	48.08	N/A	N/A	89.91
Amantadine	1.91	26.02	11	151.25	1	1	0	45.09	287.44	0.42	65.15
Apomorphine	2.85	43.70	20	267.33	3	2	0	77.99	153.32	1.04	66.21
Atuzaginstat	2.86	81.42	27	386.41	4	2	10	93.61	352.37	0.69	71.62
Baclofen	1.86	63.32	14	213.66	2	2	4	55.50	414.85	0.09	89.13
Benserazide	-1.76	148.07	18	257.25	7	7	5	61.48	1825.70	0.74	85.47
Benzatropine	4.42	12.47	23	307.44	2	0	4	93.41	341.43	0.11	74.54
Biperiden	3.96	23.47	23	311.47	2	1	5	94.09	347.75	0.02	73.52
Blarcamesine	3.53	12.47	21	281.40	2	0	4	86.28	159.46	-0.10	81.46
Brexpiprazole	4.72	48.57	31	433.58	5	1	7	129.78	588.07	0.64	66.08
Bromocriptine	3.19	118.21	43	654.61	6	3	5	164.16	58.41	0.05	4.53
Cabergoline	3.19	71.68	33	451.62	4	2	8	132.37	573.24	0.60	39.00
Caffeine	-1.03	61.82	14	194.19	6	0	0	51.20	222.13	0.19	79.68
Carbidopa	-0.05	115.81	16	226.23	5	5	4	56.84	1996.23	0.73	79.61
Curcumin	3.37	93.06	27	368.39	6	2	8	102.02	1411.26	0.07	74.57
Dantrolene	1.74	118.05	23	314.26	6	1	4	78.64	520.79	0.77	76.26
Donepezil	4.36	38.77	28	379.50	4	0	6	110.13	716.51	0.23	72.29
Eicosapentaenoic Acid	6.47	26.30	24	330.51	2	0	14	104.94	9700.47	0.74	88.40

Entacapone	1.78	127.70	22	305.29	6	2	5	77.94	1009.98	0.97	81.65
Escitalopram	3.81	36.26	24	324.40	3	0	5	90.91	688.70	0.48	68.02
Fingolimod	3.20	66.48	22	307.48	3	3	12	92.93	5986.27	0.14	86.39
Galantamine	1.85	41.93	21	287.36	4	1	1	79.80	490.38	0.46	46.33
Guanfacine	1.55	78.97	15	246.10	2	3	2	60.22	845.71	0.80	87.53
Hydralazine	0.92	63.83	12	160.18	4	2	1	47.35	315.46	0.89	83.09
Istradefylline	2.12	80.28	28	384.44	8	0	6	109.06	1642.55	0.41	70.05
Ladostigil	2.35	41.57	20	272.35	3	1	4	78.67	215.22	0.27	69.78
L-DOPA	0.05	103.78	14	197.19	4	4	3	49.09	2624.71	0.26	81.53
Levitiracetam	-0.13	63.40	12	170.21	2	1	3	44.22	2412.72	0.20	83.72
Lisuride	2.84	51.37	25	338.46	2	2	3	101.64	343.15	0.69	49.96
Masitinib	5.26	73.39	36	498.66	7	2	7	146.98	2844.94	0.43	66.10
Melatonin	1.86	54.12	17	232.28	2	2	4	67.24	1913.49	0.08	78.11
Memantine	2.69	26.02	13	179.31	1	1	0	54.32	277.49	0.19	62.45
Metformin	-1.03	88.99	9	129.17	2	4	0	36.46	156.48	0.21	93.69
Modafinil	2.01	60.16	19	273.36	2	1	5	76.93	2301.01	N/A	84.62
Nilotinib	6.36	97.62	39	529.53	7	2	6	140.98	N/A	0.38	62.79
Opicapone	3.28	149.46	27	413.17	8	2	3	93.61	1772.59	0.46	74.81
Pimavanserin	4.67	44.81	31	427.56	3	1	8	121.47	645.60	0.22	73.81
Piribedil	1.53	50.72	22	298.35	6	0	3	82.09	486.40	0.10	76.33
Pramipexole	1.58	50.94	14	211.33	4	2	3	60.63	1980.96	0.41	74.35
Procyclidine	3.94	23.47	21	287.45	2	1	5	87.20	395.47	-0.01	82.08

Rasagiline	1.90	12.03	13	171.24	1	1	2	54.34	279.24	0.27	75.60
Remacemide	2.22	55.12	20	268.36	2	2	5	81.14	870.47	0.15	83.58
Riluzole	2.78	48.14	15	234.20	4	1	1	50.72	173.51	0.72	78.46
Rivastigmine	2.76	32.78	18	250.34	3	0	4	72.87	389.57	0.66	79.66
Ropinirole	2.85	32.34	19	260.38	2	1	7	79.50	454.08	0.50	78.40
Rotigotine	4.27	23.47	22	315.48	3	1	6	93.81	702.34	0.29	70.26
Selegiline	2.18	3.24	14	187.29	1	0	4	61.07	311.03	0.67	83.80
Simufilam	1.04	35.58	19	259.35	3	1	2	74.46	524.01	0.19	79.94
Tacrine	2.70	38.91	15	198.27	2	1	0	62.80	1094.28	0.96	76.33
Tetrabenazine	3.24	38.77	23	317.43	4	0	4	90.13	554.92	0.32	69.21
Tizanidine	1.72	62.20	16	253.72	6	2	1	66.35	424.51	0.33	79.74
Tolcapone	2.55	100.67	20	273.24	5	2	3	71.04	2956.93	0.65	82.74
Trihexyphenidyl	4.33	23.47	22	301.47	2	1	5	91.82	519.12	0.21	81.82
Valiltramiprosate	-0.64	109.49	15	238.31	4	3	6	56.86	1156.59	0.38	82.60
Average	2.42	60.39	20.81	291.39	3.67	1.52	4.30	81.34	1142.87	0.41	74.38
Maximum	6.47	149.46	43.00	654.61	8.00	7.00	14.00	164.16	9700.47	1.04	93.69
Minimum	-1.76	3.24	9.00	129.17	1.00	0.00	0.00	36.46	58.41	-0.10	4.53
SD*	1.79	35.24	7.15	100.70	1.90	1.37	2.92	27.96	1601.32	0.29	14.23

* Standard deviation.

Selection Score (S^S) and elimination scores (S^E)

The S^S give a weight to each designed caffeic acid derivative (dCAF) depending on whether they meet the physicochemical parameters and evaluate their ADME properties (S^{ADME}), as well as whether they are non-toxic (S^T) and accessible to be synthesized (S^{SA}). Hence, the following formulations have been proposed to obtain the selection scores.

$$\boldsymbol{S^S} = 0.4 * S^{ADME} + 0.4 * S^T + 0.2 * S^{SA}$$

where

$$\boldsymbol{S^{ADME}} = \frac{S^{logP} + S^{HBD} + S^{HBA} + S^{MW} + S^{MR} + S^{AtX} + S^{RB} + S^{PSA}}{8}$$

Where the score will be 1 or 0 depend of:

$$S^{logP} = \begin{cases} 1, if\ -0.4 \leq logP \leq 5.0 \\ 0, otherwise \end{cases} \quad S^{HBD} = \begin{cases} 1, if\ HBD \leq 5 \\ 0, otherwise \end{cases} \quad S^{HBA} = \begin{cases} 1, if\ HBA \leq 10 \\ 0, otherwise \end{cases}$$

$$S^{MW} = \begin{cases} 1, if\ 160 \leq MW \leq 480 \\ 0, otherwise \end{cases} \quad S^{MR} = \begin{cases} 1, if\ 40 \leq MR \leq 130 \\ 0, otherwise \end{cases} \quad S^{AtX} = \begin{cases} 1, if\ AtX \leq 70 \\ 0, otherwise \end{cases}$$

$$S^{RB} = \begin{cases} 1, if\ RB \leq 10 \\ 0, otherwise \end{cases} \quad S^{PSA} = \begin{cases} 1, if\ PSA \leq 140 \\ 0, otherwise \end{cases}$$

$$\boldsymbol{S^T} = \frac{S^{LD_{50}} + S^M}{2}$$

$$S^{LD_{50}} = 1 + log\left(\frac{{LD_{50}}^{dCAF}}{{LD_{50}}^{\overline{RefSet}}}\right) \quad S^M = 1 - M^{dCAF}$$

$$\boldsymbol{S^{SA}} = 1 + log\left(\frac{SA^{dCAF}}{SA^{\overline{RefSet}}}\right)$$

To verify if any designed caffeic acid derivative deviates significantly from the average value of the reference set, in any of the properties analyzed, the elimination scores (S^E) were used:

$$S^{E,logP} = \left|\frac{logP_{\overline{RefSet}} - logP_{dCAF}}{SD_{logP}}\right| \qquad S^{E,HBD} = \left|\frac{HBD_{\overline{RefSet}} - HBD_{dCAF}}{SD_{HBD}}\right| \qquad S^{E,HBA} = \left|\frac{HBA_{\overline{RefSet}} - HBA_{dCAF}}{SD_{HBA}}\right| \qquad S^{E,MW} = \left|\frac{MW_{\overline{RefSet}} - MW_{dCAF}}{SD_{MW}}\right|$$

$$S^{E,MR} = \left|\frac{MR_{\overline{RefSet}} - MR_{dCAF}}{SD_{MR}}\right| \qquad S^{E,AtX} = \left|\frac{AtX_{\overline{RefSet}} - AtX_{dCAF}}{SD_{AtX}}\right| \qquad S^{E,RB} = \left|\frac{RB_{\overline{RefSet}} - RB_{dCAF}}{SD_{RB}}\right| \qquad S^{E,PSA} = \left|\frac{PSA_{\overline{RefSet}} - PSA_{dCAF}}{SD_{PSA}}\right|$$

$$S^{E,LD_{50}} = \left|\frac{LD_{50}{}_{\overline{RefSet}} - LD_{50}{}_{dCAF}}{SD_{LD_{50}}}\right| \qquad S^{E,M} = \left|\frac{M_{\overline{RefSet}} - M_{dCAF}}{SD_{M}}\right| \qquad S^{E,SA} = \left|\frac{SA_{\overline{RefSet}} - SA_{dCAF}}{SD_{SA}}\right|$$

It is important to note that larger values of these scores may represent better or worse performance than the average of the reference drugs. For example, in the case of $S^{E,LD_{50}}$, if the LD_{50} of a dCAF is bigger than the LD_{50} of Reference set, it indicates that the dCAF is better because it is less toxic.

Table S4. Values of the ADME properties, toxicity and synthetic accessibility for the twenty designed caffeic acid derivatives (dCAF) with higher S^S. Oral rat 50 percent lethal dose (LD_{50}), Ames mutagenicity (M) and synthetic accessibility (SA). log P, polar surface area (PSA), number of non-hydrogen atoms (AtX), molecular weight (MW), number of acceptors in H-bridge interactions (HBA), number of donors in H-bridge interactions (HBD), number of rotatable bonds (RB), molar refractivity (MR). Also the S^S is presented.

	logP	PSA	AtX	MW	HBA	HBD	RB	MR	LD_{50}	M	SA	S^S
CAF	1.20	77.76	13.00	180.16	3.00	3.00	2.00	46.44	2395.16	0.24	87.46	1.030
dCAF-16	0.74	104.06	17.00	238.19	5.00	3.00	3.00	57.40	5573.84	0.06	84.38	1.137
dCAF-82	1.09	107.22	16.00	226.18	5.00	4.00	3.00	54.57	5909.51	0.12	84.83	1.130
dCAF-2	1.08	97.99	14.00	196.16	4.00	4.00	2.00	48.01	4738.91	0.04	86.00	1.128
dCAF-108	0.91	107.22	16.00	226.18	5.00	4.00	3.00	54.66	5966.11	0.14	85.30	1.127
dCAF-80	1.09	107.22	16.00	226.18	5.00	4.00	3.00	54.57	5464.77	0.13	85.10	1.122
dCAF-17	0.74	104.06	17.00	238.20	5.00	3.00	3.00	57.40	3855.15	0.02	83.89	1.120
dCAF-1076	0.90	118.22	16.00	230.15	5.00	5.00	2.00	49.82	3321.30	0.05	84.98	1.114
dCAF-25	1.20	86.99	15.00	210.18	4.00	3.00	3.00	52.99	4540.90	0.09	85.69	1.114
dCAF-149	0.91	107.22	16.00	226.18	5.00	4.00	3.00	54.66	5001.87	0.13	85.23	1.114
dCAF-157	1.92	97.99	18.00	264.15	4.00	4.00	2.00	53.11	2709.32	0.14	82.91	1.112
dCAF-525	1.11	118.22	16.00	230.15	5.00	5.00	2.00	49.54	6034.05	0.22	85.12	1.112
dCAF-527	1.11	118.22	16.00	230.15	5.00	5.00	2.00	49.54	6034.05	0.22	84.55	1.112
dCAF-148	0.88	107.22	16.00	226.18	5.00	4.00	3.00	54.06	4243.79	0.08	84.83	1.109
dCAF-315	1.31	86.99	16.00	228.17	4.00	3.00	3.00	52.35	3851.50	0.04	85.06	1.109
dCAF-1441	2.09	97.99	19.00	296.22	5.00	5.00	2.00	61.03	10059.44	0.45	82.15	1.108
dCAF-19	0.98	104.06	17.00	238.19	5.00	3.00	3.00	57.78	5065.65	0.17	84.69	1.107
dCAF-1094	0.90	118.22	16.00	230.15	5.00	5.00	2.00	49.82	2970.68	0.06	84.79	1.106
dCAF-20	0.98	104.06	17.00	238.19	5.00	3.00	3.00	57.78	3268.12	0.02	84.07	1.106
dCAF-23	1.20	86.99	15.00	210.18	4.00	3.00	3.00	52.99	3740.09	0.05	86.07	1.106
dCAF-1365	1.34	97.99	16.00	232.14	4.00	4.00	2.00	48.12	2910.47	0.06	84.87	1.105

Table S5. Elimination scores (S^E) for the subset of twenty designed caffeic acid derivatives (dCAF) chosen as the most promising, according to S^S.

	$S^{E,logP}$	$S^{E,PSA}$	$S^{E,AtX}$	$S^{E,MW}$	$S^{E,HBA}$	$S^{E,HBD}$	$S^{E,RB}$	$S^{E,MR}$	$S^{E,LD50}$	$S^{E,M}$	$S^{E,SA}$
CAF	0.682	0.493	1.093	1.105	0.350	1.081	0.787	1.248	0.782	0.596	0.919
dCAF-16	0.938	1.239	0.534	0.528	0.701	1.081	0.444	0.856	2.767	1.224	0.702
dCAF-82	0.743	1.329	0.674	0.648	0.701	1.811	0.444	0.957	2.977	1.014	0.734
dCAF-2	0.748	1.067	0.953	0.946	0.175	1.811	0.787	1.192	2.246	1.293	0.816
dCAF-108	0.843	1.329	0.674	0.648	0.701	1.811	0.444	0.954	3.012	0.945	0.767
dCAF-80	0.743	1.329	0.674	0.648	0.701	1.811	0.444	0.957	2.699	0.980	0.753
dCAF-17	0.938	1.239	0.534	0.528	0.701	1.081	0.444	0.856	1.694	1.503	0.668
dCAF-1076	0.849	1.641	0.674	0.608	0.701	2.541	0.787	1.127	1.360	1.607	0.744
dCAF-25	0.682	0.755	0.813	0.806	0.175	1.081	0.444	1.014	2.122	1.119	0.794
dCAF-149	0.843	1.329	0.674	0.648	0.701	1.811	0.444	0.954	2.410	0.980	0.762
dCAF-157	0.280	1.067	0.394	0.271	0.175	1.811	0.787	1.010	0.978	1.921	0.599
dCAF-525	0.732	1.641	0.674	0.608	0.701	2.541	0.787	1.137	3.054	0.666	0.755
dCAF-527	0.732	1.641	0.674	0.608	0.701	2.541	0.787	1.137	3.054	0.666	0.714
dCAF-148	0.860	1.329	0.674	0.648	0.701	1.811	0.444	0.976	1.936	1.154	0.734
dCAF-315	0.620	0.755	0.674	0.628	0.175	1.081	0.444	1.037	1.691	1.293	0.750
dCAF-1441	0.185	1.067	0.254	0.048	0.701	2.541	0.787	0.726	5.568	0.136	0.546
dCAF-19	0.804	1.239	0.534	0.528	0.701	1.081	0.444	0.843	2.450	0.840	0.724
dCAF-1094	0.849	1.641	0.674	0.608	0.701	2.541	0.787	1.127	1.141	1.642	0.731
dCAF-20	0.804	1.239	0.534	0.528	0.701	1.081	0.444	0.843	1.327	1.503	0.681
dCAF-23	0.682	0.755	0.813	0.806	0.175	1.081	0.444	1.014	1.622	1.259	0.821
dCAF-1365	0.603	1.067	0.674	0.588	0.175	1.811	0.787	1.188	1.104	1.642	0.737

Table S6. Reactivity indexes, their acronyms, calculation method and interpretation.

	Acronym	Calculation	Interpretation
First ionization energy (eV)	IE	ΔSCF<br>$E(N-1)-E(N)$	Directly related to the capability of donating one electron. The lower the IE the most likely the antioxidant protection, via electron transfer.
Bond dissociation energies (kcal/mol)	BDE	$ZPE(D)+ZPE(H)$<br>$-ZPE(DH)$	Measures the energy necessary for breaking donor(D)-H bonds. The lower the BDE, the higher the antioxidant activity, via H transfer.

D: Caffeic acid derived. N: System electron number.

Table S7. First ionization energy (IE, eV) and the lowest bond dissociation energies (*l*-BDE, kcal/mol) for the designed caffeic acid derivatives (dCAF) chosen as the most promising, according to S^S.

Neutral	IE	*l*-BDE
CAF	5.96	81.66
dCAF-2	6.02	82.88
dCAF-16	6.06	83.29
dCAF-82	5.65	78.36
Mono-anion		
CAF	5.75	79.49
dCAF-2	6.11	80.85
dCAF-16	5.89	80.63
dCAF-82	5.54	77.23
Di-anion		
CAF	4.67	75.14
dCAF-2	4.74	75.01
dCAF-16	4.81	76.09
dCAF-82	4.65	72.46
Reference Antioxidants		
Trolox (Mono-anion)	5.23	77.78

Table S8. Tunneling values for the reactions of dCAF-2, dCAF-16 y dCAF-82 with •OOH, in water (W) and pentylethanoate (PE), at 298.15 K.

	PE	Water		
	Neutral	Neutral	Mono-anion	Di-anion
dCAF-2				
f-HAT				
O7a	57.12	241.28	129.28	na
O8a	337.86	477.58	162.97	barrier-less
dCAF-16				
f-HAT				
O7a	824.41	995.25	371.96	na
O8a	118.09	243.36	138.08	barrier-less
dCAF-82				
f-HAT				
O7a	16.08	535.37	260.32	na
O8a	12.48	17.56	12.24	barrier-less
